# Frequency dynamics predict viral fitness, antigenic relationships and epidemic growth

**DOI:** 10.1101/2024.12.02.24318334

**Published:** 2025-09-12

**Authors:** Marlin D. Figgins, Trevor Bedford

**Affiliations:** 1 Vaccine and Infectious Disease Division, Fred Hutchinson Cancer Center, Seattle, WA, USA,; 2 Department of Applied Mathematics, University of Washington, Seattle, WA, USA,; 3 Howard Hughes Medical Institute, Seattle, WA, USA,

## Abstract

During the COVID-19 pandemic, SARS-CoV-2 variants drove large waves of infections, fueled by increased transmissibility and immune escape. Current models focus on changes in variant frequencies without linking them to underlying transmission mechanisms of intrinsic transmissibility and immune escape. We introduce a framework connecting variant dynamics to these mechanisms, showing how host population immunity interacts with viral transmissibility and immune escape to determine relative variant fitness. We advance a selective pressure metric that provides an early signal of epidemic growth using genetic data alone, crucial with current underreporting of cases. Additionally, we show that a latent immunity space model approximates immunological distances, offering insights into population susceptibility and immune evasion. These insights refine real-time forecasting and lay the groundwork for research into the interplay between viral genetics, immunity, and epidemic growth.

## Introduction

The COVID-19 pandemic was marked by the successive emergence of SARS-CoV-2 variant viruses, driving repeated epidemics globally [1, 2]. While these repeated large waves occurred with the emergence of novel variants, the mechanism driving these variants’ success changed over time. The spread of early variants such as Alpha, Beta, Gamma and Delta were largely driven by increases in intrinsic transmissibility [3]. The Omicron variant showed substantial immune escape [3] and subsequent derived lineages within Omicron including XBB, EG.5.1 and JN.1 appear to be driven by immune escape as evidenced through molecular studies of neutralization using human sera [4–7]. Since 2022, there has been repeated replacement by subsequent Omicron-derived lineages. This rapid viral population turnover is consistent with antigenic evolution and is observed in other viruses such as seasonal influenza [8], although SARS-CoV-2 currently remains an outlier in terms of pace of its evolution [9]. This transition from transmissibility-driven to immune escape-driven success is a consequence of the interplay between population immunity and variant fitness.

With the increased temporal and geographical scale of sequencing alongside a detailed genetic nomenclature [10] and bioinformatic tools for lineage assignment [11, 12], we have gained more data for SARS-CoV-2 than for other circulating viruses giving a unique opportunity for insight into its evolution. Several models of variant frequency have been developed to estimate the fitness of emerging SARS-CoV-2 variants [13–18]. These models estimate the relative fitness (or selective advantage) of circulating variant viruses from their frequency in sequencing data, typically represented by counts of variant sequences over time within a geographic region. Relative fitness in these models is often assumed to be constant and intrinsic to the variant of interest. However, this may be an oversimplification of the transmission process.

It has been shown that these transmission advantages differ geographically and temporally, suggesting that variant transmission advantages are not necessarily fixed and may be informed by regional population differences [15, 19]. In fact, heterogeneity in transmission advantages may be well explained by regional differences in immune structure as Dadonaite et al. [20] show deep mutational scanning estimates of immune escape are well correlated with estimated variant growth advantages. Existing models that allow variant transmission advantages to change in time often do not have a mechanistic underpinning for why transmission advantages exist and vary geographically and temporally [15, 16]. Models that do include mechanistic grounding of transmission based on population immunity such as Meijers et al. [21] and Raharinirina et al. [22] have parameterized variant-specific immunity based on serological measurements or deep mutational scanning datasets. Timely serological data is thus a requirement for these models to perform well for real-time evolutionary forecasting.

**Fig. 1.**
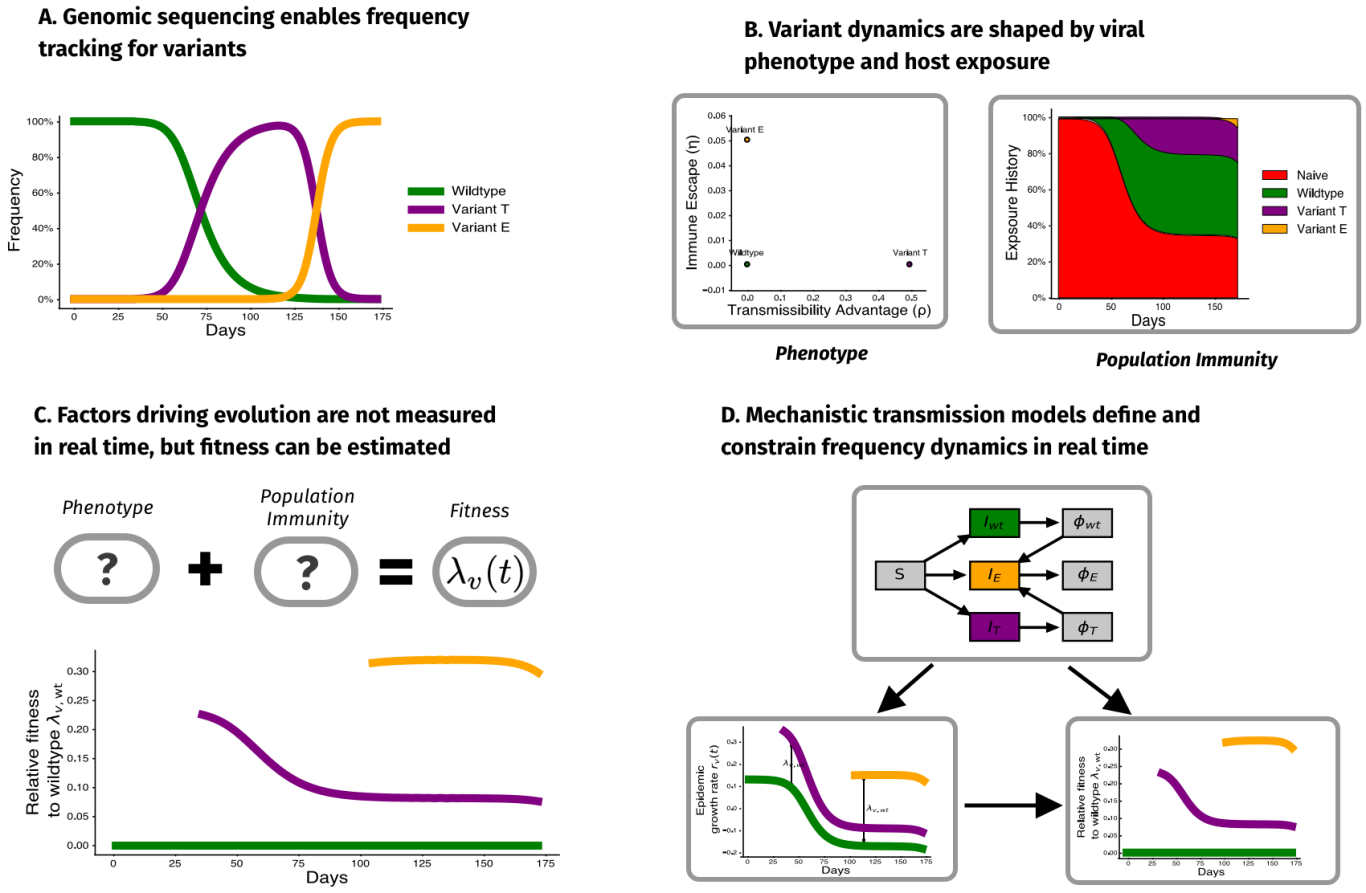
Mechanistic transmission models inform frequency dynamics. (A) Genomic surveillance reveals changes in the frequency of genetic variations over time. (B) These frequency changes can arise due to differences in phenotypes related to transmission (e.g, immune escape, transmissibility, binding) and changes in population immunity due to recent exposure. (C) Despite being instrumental for real-time analysis, both variant phenotype and population immunity are rarely observed in real time. (D) We use mechanistic transmission models to infer relative fitness from frequency data alone, taking advantage of known structure in transmission dynamics. This enables us to quantify trade-offs between variant phenotypes and develop new methods for estimating fitness in populations undergoing antigenic evolution.

In response to this gap, we introduce a novel framework that links variant dynamics directly to transmission mechanisms using compartmental models of infectious diseases (Fig. 1). By modeling both intrinsic transmissibility and immune escape, we explain how shifts in population immunity shape the relative fitness of viral variants and select for immune escape over intrinsic transmissibility with increasing past exposure. Furthermore, including these mechanisms suggests that relative fitness varies in time, reflecting the evolving landscape of population immunity and exposure regardless of the underlying mechanism.

Here, we present a novel non-parametric method for estimating time-varying fitness regardless of the underlying transmission mechanism. Alongside this development we introduce a “selective pressure” metric that quantifies the impact of variant turnover on population-level epidemic growth rates. Finally, we develop a latent immunity model that we use to estimate the underlying proportion of pseudo-immune groups within multiple geographies and pseudo-immune escape rates for circulating variants that predicts antigenic distances using sequence data alone. Overall, our framework bridges the gap between genetic data and transmission dynamics, offering a new way to predict and manage viral outbreaks.

## Results

### Variant dynamics and relative fitness in multistrain models

Multi-strain models of epidemics have been developed to understand the competition between different viral strains that exhibit different levels of cross-immunity [23, 24]. These models have typically been used to explain strain evolution in antigenically variable pathogens like seasonal influenza virus [8] and seasonal coronaviruses [25, 26].

We begin by modeling a population of V exponentially growing variant viruses each with prevalence $I_{v}(t)$ and time-varying growth rate $r_{v}(t)$. By considering the difference in these growth rates, we can define the relative fitness as $\lambda_{v,u}(t)=r_{v}(t)-r_{u}(t)$. This relative fitness determines the change in the frequencies of the variants in the population

(1) $$f_{v}\left(t\right)=\frac{f_{v}\left(0\right)\text{exp}\left(\int_{0}^{t}\lambda_{v,v^{*}}\left(s\right)ds\right)}{\sum_{u=1}^{V}f_{u}\left(0\right)\text{exp}\left(\int_{0}^{t}\lambda_{u,v^{*}}\left(s\right)ds\right)},$$

where $v^{*}$ is a chosen pivot variant that has relative fitness zero.

In order to better understand frequency dynamics of pathogens with multiple co-circulating variants, we apply the above framework to compartmental models of epidemics, which can be written as time-varying exponential growth (detailed in Supplementary Text S1). These models provide an intuition of how strain-level selection depends on the assumed transmission mechanism of the underlying epidemic model. This framework also generalizes several existing methods for relative fitness estimation and prediction (detailed in Supplementary Text S2). We summarize dynamics of a three-variant mechanistic transmission model in Fig. S1, where we compare a transmission variant T with a 50% increase in transmissibility (ρ=0.5) to an escape variant E that infects 5% of hosts possessing wildtype immunity (η=0.05).

Our approach shows that relative fitness is often dependent on the past exposure of a population (as discussed in Supplementary Text S1 and extended to full immune history models in Supplementary Text S3). This suggests that serology, vaccination history, and immunological data generally can be informative of relative fitness. Additionally, when working with variant classifications, non-neutral evolution within a variant will cause the relative fitness of that variant to change in time. However, even in the absence of external data that can inform relative fitness, there is still hope.

We develop a method for using approximate Gaussian processes to model variant relative fitness. Gaussian processes are probability distributions over functions, where the structure and smoothness of these functions are defined by a kernel that encodes correlations in time. These models are flexible and allow us to encode smoothness constraints, periodicity, and other structures [27]. Gaussian processes allow us a non-parametric estimate of the relative fitness for variants through time (see Materials and Methods).

Traditional Gaussian processes, while flexible, face challenges for large time series and large data sets. Our approach overcomes this using a Hilbert Space Gaussian Process (HSGP) approximation, making the framework scalable for many variants and long time periods [28]. This enables real-time variant fitness estimation and can be applied to any frequency data regardless of the underlying mechanism. This model is used in Fig. S2 to estimate the relative fitnesses of different variants through time based on simulated variant sequence counts from frequencies shown in Fig. S1.

Later, we apply this model to empirical SARS-CoV-2 sequence data from 50 US states and England from 2021 to 2022 to estimate relative fitness for variants circulating in that period, but first we continue analytic investigation into fitness dynamics.

### Determining the transmissibility-escape tradeoff

To understand the fitness trade-off between transmissibility and immune escape, we consider dynamics with a wildtype virus W with $\rho_{W}=0$ and $\eta_{W}=0$, an increased transmissibility variant T with $\rho_{T}>0$ and $\eta_{T}=0$ and an immune escape variant E with $\rho_{E}=0$ and $\eta_{E}>0$.

Following Equation 35, we write relative fitnesses of the escape variant or transmissibility variant as

(2) $$\lambda_{E,W}=\eta \beta \phi_{W}\left(t\right),$$

(3) $$\lambda_{T,W}=\rho \beta S\left(t\right),$$

where β is the transmissibility coefficient, η is escape proportion against the wildtype and ρ is the variant’s proportional increase to transmissibility.

In the simplest case where individuals are either susceptible or have wildtype immunity $\left(S(t)+\phi_{W}(t)=1\right)$, we can compute the critical immune fraction $\phi^{*}$ at which $\lambda_{E,W}\left(\phi^{*}\right)=\lambda_{T,W}\left(\phi^{*}\right)$ as

(4) $$\phi^{*}=\frac{\rho }{\eta +\rho }.$$

**Fig. 2.**
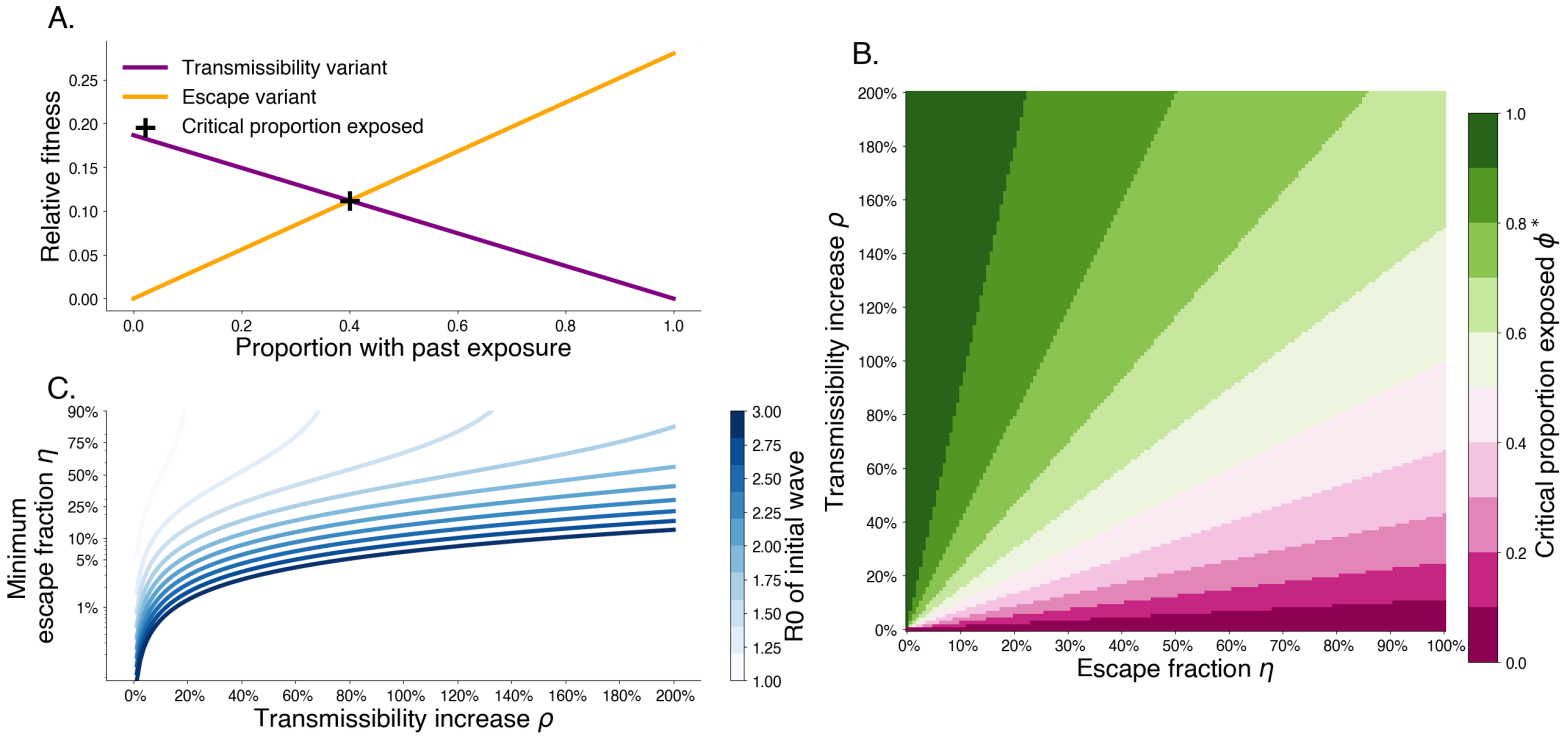
Trade-off between degree of immune escape and increased transmissibility. A. Relative fitness for a transmissibility increasing variant T with $\rho_{T}=0.2$ and an immune escaping variant E with $\eta_{E}=0.3$ for $R_{0,W}=2.8$ and 1/γ=3.0 days. The intersection point shows that after 40% of the population has wildtype immunity, the escape variant has higher fitness. B. The critical exposure proportion is shown for various escape fraction and transmissibility increase. Above the critical exposure proportion, we expect dominance of escape variants. C. The minimum escape fraction needed for second waves to be comprised of escape variant assuming competition with transmissibility increase variants and first wave with a given $R_{0}$.

For past exposure level greater than $\phi^{*}$ escape variants have a higher relative fitness. This trade off shows that increasing degree of escape entails that a lower proportion of past exposure is needed for escape variants to be preferred (Fig. 2). Additionally, this shows that when intrinsic transmissibility increases are limited escape is more likely to be a dominant mechanism for variant turnover.

### Initial growth rates insufficient for predicting short-term frequency growth

One question of interest is whether knowledge of mechanism meaningfully informs our ability to forecast short-term frequency growth. The first step to addressing this is to understand how the relative fitness may change in time to understand the predictability of relative fitness in the short-term.

We find that the mechanistic forms analyzed in this paper (Supplementary Text S1) can be represented as weighted combinations of B time-varying functions $\Upsilon_{b}(t)$ with weights $\beta_{b}$. We can think of each of these functions $\Upsilon_{b}$ as an immune background and the coefficient $\beta_{b}$ as a transmission differential, so that

(5) $$\lambda_{v,u}\left(t\right)=\sum_{1\leq b\leq B}\beta_{b}\Upsilon_{b}\left(t\right).$$

Even in the case of complete knowledge of the relative fitness and the underlying fitness contributions in the present and past, we have that change in the relative fitness is determined by

(6) $$\frac{d\lambda_{v,u}}{dt}=\sum_{1\leq b\leq B}\beta_{b}\frac{d\Upsilon_{b}}{dt}\left(t\right).$$

By considering a Taylor expansion of the relative fitness about the point of estimation $t_{0}$, we can approximate the relative fitness in the future as

(7) $$\lambda_{v,u}\left(t\right)\approx \lambda_{v,u}\left(t_{0}\right)+\left(t-t_{0}\right)\sum_{1\leq b\leq B}\beta_{b}\frac{d\Upsilon_{b}}{dt}\left(t_{0}\right).$$

This suggests small differences in the form of $\lambda_{v,u}(t)$ can lead to meaningful differences in the future relative fitnesses through changes in the underlying immune backgrounds.

We investigate whether relative fitnesses vary predictably in the short-term regardless of mechanism. To do so, we apply the two-variant model developed in previous sections for different mechanisms of immune escape and increased transmissibility. We fix the relative fitness of the novel variant at a prediction time $t_{0}$ using Equation 4 and assess the change in the relative fitness in the short-term. We find that although relative fitness trajectories share the same decreasing shape, they may decline at different rates depending on the mechanism (Fig. S3). This can lead to substantial changes in the predicted incidence depending on the assumed mechanism and affects to overall rate of turnover.

### Correlations insufficient for mechanism identification

Although correlations between vaccination uptake and variant growth advantage are often observed, these alone may not be sufficient to identify the mechanism behind a variant’s success. A variant’s fitness advantage may arise from increased transmissibility, immune escape, or a combination of both. Even in the absence of immune escape, the relative fitness of a variant depends on the proportion of the population that is susceptible to infection and therefore changes with both past exposure and vaccine uptake (Supplementary Text S1). To illustrate this, we simulate the spread of a variant with increased transmissibility in populations with varying initial vaccination levels.

**Fig. 3.**
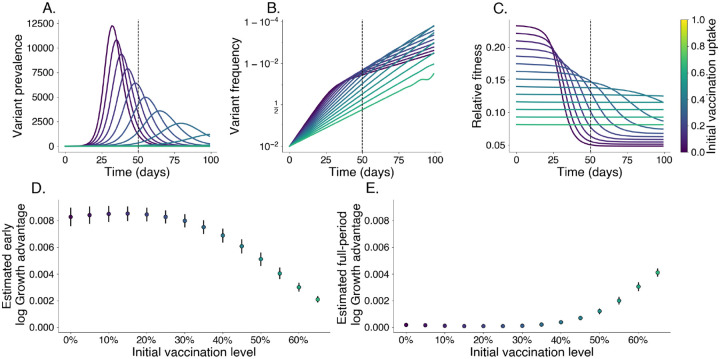
Relative fitness is correlated with vaccination levels in the absence of immune escape. We simulate the growth of a pure transmissibility increased variant at varying levels of vaccination. Darker colors represent lower vaccine uptake. We identify an early growth period where relative fitness is at its highest; the cutoff for this period is denoted with a vertical dashed line. A. Prevalence of variant, each line is its own simulation. B. Frequency of variant. C. Relative fitness for variant over time. D. Estimated log growth advantage using linear regression of log relative frequency of variant over wildtype using only data before the early cutoff. E. Same as D. but using data from the entire period shown.

In populations with lower vaccination levels, the variant’s prevalence peaks more sharply and its relative fitness declines quickly as immunity accumulates within the population (Fig. 3A–C). In contrast, higher vaccination levels constrain relative fitness, leading to a delayed peak in prevalence and more stable relative fitness as the existing immunity limits the variant’s spread (Fig. 3A–C). Even without immune escape, estimated growth advantages for this variant decrease with increasing vaccination uptake near the beginning of an epidemic (Fig. 3D). Later in the epidemic, this relationship reverses with estimated growth advantages over the full period increasing with initial vaccination levels, which may be mistaken as signal for immune escape (Fig. 3E).

This analysis shows that correlation-based methods alone may struggle to identify the true mechanisms driving a variant’s success especially under the assumption of a fixed growth advantage. By explicitly considering how immunity and transmissibility interact within populations, models that incorporate these dynamics may provide a stronger foundation for understanding why certain variants spread.

### Quantifying selective pressure

Although it is useful to quantify the relative fitnesses of individual variants, we are often interested in quantifying the overall effects of selection in the population. With this in mind, we can derive a metric of overall selective pressure

(8) $$\psi (t)=E_{f(t)}\left[\frac{d\lambda_{v}}{dt}\right]+V_{f(t)}\left[\lambda_{v}\right]$$

that describes the distribution of relative fitness in the population. This selective pressure metric serves as an indicator for high fitness variants arising in the population as change. High fitness variants rising from initially low frequency leads to large increases in the variance of the fitness distribution and therefore increases in the selective pressure.

The selective pressure metric enables us to decompose changes in the average growth rate in the population, $d\overset{‾}{r}/dt$, to an evolutionary component ψ and a residual baseline growth rate $r_{W}$ following

(9) $$\frac{d\overset{‾}{r}}{dt}=\frac{dr_{W}}{dt}+\psi \left(t\right).$$

This shows that increased selective pressure through emerging high fitness variants can drive waves of infection. Further, this suggests that differences between growth rates based on selective pressure alone and observed rates are attributable to changes in baseline transmission over time. This mirrors ideas of Fisher’s theorem of natural selection and its later interpretations with the variance of fitness contributing directly to the change in transmission rates (or fitness) [29, 30]. This definition of selective pressure captures how relative fitness contributes to epidemic growth. This is similar to ideas quantifying rates of adaptation via fitness flux [31].

In this case, the overall growth rate $\overset{‾}{r}$ and relative incidence I(t)/I(0) can be written directly

(10) $$\overset{‾}{r}\left(t\right)=\overset{‾}{r}\left(0\right)+\left[r_{W}\left(t\right)-r_{W}\left(0\right)\right]+\Psi \left(t\right),$$

(11) $$\frac{I(t)}{I(0)}=\text{exp}\left(\int_{0}^{t}\left[r_{W}\left(s\right)+\Psi \left(s\right)\right]ds\right),$$

using the cumulative selective pressure $\Psi (t)=\int_{0}^{t}\psi (s)ds$. In addition to estimating the relative fitness, metrics derived from these models can inform us of much more.

Our “selective pressure” metric allows us to model the contribution of evolution to changes in the epidemic growth rate of a population and is independent of pivot choice for relative fitness estimation. This metric acts as an early warning system for variant-driven outbreaks, especially in scenarios where case data are sparse or delayed. This metric can be computed using any method that estimates variant frequency and relative fitnesses and serves as a simple tool for understanding the contribution of selection to the overall population dynamics.

The full derivation of this metric and its contribution to the overall growth rate can be found in Supplementary Text S4.

### Predicting epidemic growth rates using selective pressure

**Fig. 4.**
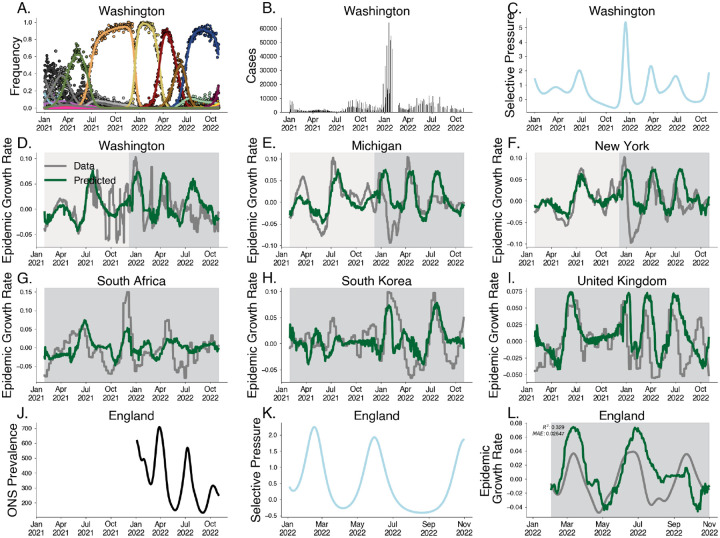
Predicting epidemic growth rate using estimated selective pressure. A. Variant frequency estimated using the Gaussian process relative fitness model between January 2021 and November 2022 for sequence count data from Washington state. B. Case counts from Washington state. C. Selective pressure computed using estimated variant frequencies and relative fitnesses from Washington state. D-F. Predictions for empirical growth rate from selective pressure for selected US states. The light gray period is the training period and the darker gray is the testing period. G-I. Predictions for empirical growth rate from selective pressure for countries South Africa, South Korea and the UK. J. Prevalence estimates for England from ONS Infection Survey. K. Estimated selective pressure in England. L. Empirical growth rates (gray) computed from prevalence estimates and predictions from our model (green) computed from selective pressure.

Motivated by the relationship between epidemic growth rate and selective pressure demonstrated above, we develop a predictive model of epidemic growth rate using estimates of selective pressure. Using empirical SARS-CoV-2 case and sequence data from 50 US states between January 2021 and November 2022, we estimate epidemic growth rates through time in each state using case counts, and estimate selective pressure through time using our approximate Gaussian process model on sequence counts (Fig. 4 A–C.) Here we group variants at the granularity of Nextstrain clades [12] resulting in 28 distinct variants over this time period. As expected we see that relative fitness increases through time and that selective pressure corresponds to speed of clade turnover where the sweep of Omicron BA.1 (clade 21K) yields the strongest signal of selective pressure (Figs. S4–S8). We use these estimates to fit a gradient-boosted regressor to predict epidemic growth rates using selective pressure from the most recent 28 days, reserving data between July 2022 and November 2022 for testing (Fig. 4 D–I, Fig. S9). This regressor is chosen via time series cross-validation among model architectures and grid-search parameter tuning (Fig. S10).

We observe a strong correspondence between observed epidemic growth rate and model predictions with Pearson $R^{2}$ in the training period of 0.576 and a weaker Pearson $R^{2}$ in the testing period of 0.077. As case reporting declined over this period, we expect weaker correspondence between our predictions and epidemic growth rates computed from case data. To address this, we sought to evaluate the out-of-sample fit on case data from other countries e.g. South Africa, South Korea, and the United Kingdom, achieving an $R^{2}$ of 0.196.

To address the potential for this method under steady reporting rates, we validate this method by predicting the epidemic growth rates in England derived from the Office for National Statistics (ONS) Coronavirus Infection Survey between February 2022 and November 2022. The ONS Infection Survey represented a randomly sampled panel survey of households where nasal swabs were collected regardless of symptom status allowing for prevalence estimates despite faltering case reporting [32]. Our model is able to replicate patterns seen in epidemic growth rates in England derived from ONS data (Fig. 4 J–L), achieving a coefficient of variation of $R^{2}=0.329$ and mean absolute error of 0.026. Performance is significantly better for the first two subsequent waves, falling off in accuracy for the fall 2022 BQ.1 (clade 22E) wave.

Although these predictions can be biased by non-evolutionary effects on the epidemic growth, this approach provides a simple measure of epidemic growth in the absence of high quality case counts using sequence data alone.

### Latent factor model of relative fitness

The representation of relative fitness using discrete immune backgrounds suggests that there may be low-dimensional structure to variant relative fitness. To elucidate these factors, we develop and implement a method for latent factor analysis of relative fitness from sequence data alone. This model assumes that variants intrinsically escape the immune responses with particular groups and that differences in a variant’s relative fitness between geographies is attributable to differences in immunity between populations. This enables us to estimate a pseudo-escape rates for variants as well as pseudo-immunity groups within geographies over time.

We generate Pango lineage-level sequence counts for 18 countries and 53 variants between March 2023 and March 2024. These 18 countries were chosen based on availability of sequence data. Small lineages that do not meet a count threshold are collapsed into their parent lineages. This leaves us with a total of 53 variants, so that each variant met a threshold for number of sequences available.

**Fig. 5.**
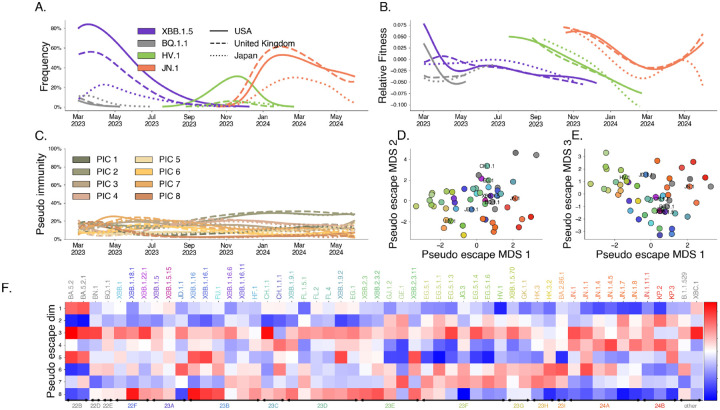
Latent factor models of immunity describe variant dynamics. We fit our latent immunity factor model for D=8 pseudo-immune groups using only SARS-CoV-2 sequence count data. A. Variant frequency. Lines are colored to show 4 variants of interest (of 53 total variants) with the style of the line denoting 3 countries of interest (of 18 total countries). B. Estimated relative fitness for selected variants and countries. These variant-specific relative fitnesses are similar across countries, but not identical. C. Estimated pseudo-immunity cohorts (PIC) over time for multiple countries ordered by decreasing share in the first geography using sequence data alone. D, E. Dimensionality-reduced pseudo-escape rates using multidimensional scaling (MDS). F. Estimated pseudo-escape rates for each variant relative to pivot variant “other”.

Using these sequence counts, we apply our latent factor model to estimate the relative fitness of each variant over time in each country, pseudo-escape rates for each variant, and pseudo-immunity for each country simultaneously for D=8 pseudo-immune groups (Fig. 5). This model is significantly constrained relative to estimating the time-varying fitness independently in each location, resulting in a model with 2,752 parameters compared to 7,488 parameters in the independent model. The results of this model are visualized in Fig. 5 for several selected variants and countries of interest.

We chose D=8 for our primary analysis by noting the point at which the loss function seems to stagnate with increasing D, i.e., the “elbow” method (Fig. S11A). Further, we observe that Bayesian Information Criterion (BIC) is minimized between 7 and 9 groups (Fig. S11D). However, the exact choice of latent immune dimensionality is necessarily somewhat arbitrary and we observe significant correlations with empirical titer data for fewer dimensions as well, although D=8 also maximizes this correlation (Fig. S11B) and its significance is maintained for all dimensions D>8 tested. Analogous figures showing pseudo-immunity and pseudo-antigenic relationships across variants can be seen for D=2 in Fig. S12, D=4 in Fig. S13, D=6 in Fig. S14 and D=10 in Fig. S15.

Our results show that closely related Pango lineages are often assigned similar pseudo-escape values. This is visible as a clustering of lineages with similar colors into similar coordinates in Fig. 5D–E suggesting our pseudo-escape values broadly align with evolutionary structure. Further, our model shows that these groups of lineages tend to target particular immune groups such as clade 24A (JN.1, JN.1.1, JN.1.4) has high pseudo-escape in dimensions 3 and 4. If immune escape is the dominant mechanism for relative fitness difference, we expect that differences in immune response between variants from serological data would mirror differences in our pseudo-escape space.

**Fig. 6.**
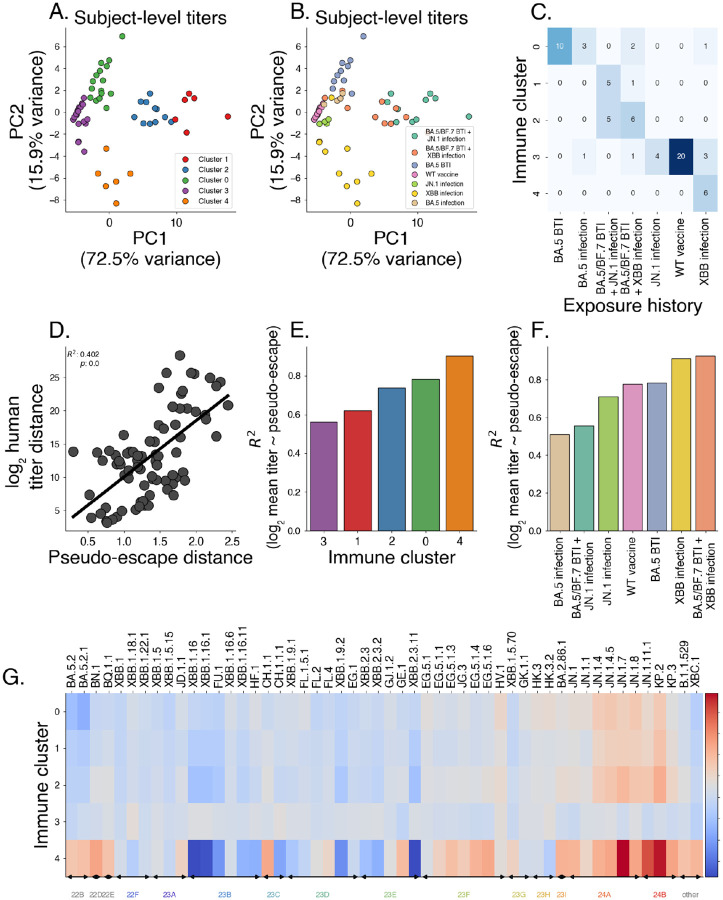
Latent factor models of immunity predict titers across varying exposure histories. Using pseudo-escape values from our latent factor model (Fig. 5) and human titer data, we show that pseudo-escape values predict antigenic distance and titers. (A-B) Principal component analysis of subject-level titers, colored by learned immune clusters (A) and subject infection history (B). (C) Co-occurrence matrix between learned immune clusters and infection histories. (D) Comparing pairwise distance between variants in the pseudo-immune space to observed distances in human titer data. (E-F) Correlation between log mean titer against a variant and that variant’s pseudo-escape by group. (G) Estimated escape burden by immune cluster, showing variant escape potential against individual immune clusters.

To examine how the learned immune structure relates to subject-level serology, we projected titers from Jian et al. [7] into principal-component space then performed clustering with k-means, arriving at k=5 clusters using the elbow method (Fig. S16). Our learned immune clusters yields clear separation (Fig. 6A) in PCA-space, whereas as a subset of subject-level exposure histories show overlap in titer measurements (Fig. 6B). This overlap can be seen in a co-occurrence matrix linking learned clusters to reported exposure histories (Fig. 6C). These groups split and aggregate multiple exposure histories. For example, individuals with a BA.5/BF.7 breakthrough infections split into both clusters 1 and 2, and cluster 3 contains individuals with XBB infection, JN.1 infection, and wildtype vaccine.

This indicates that coarse exposure histories may be an imperfect proxy for serological phenotype, and that titer-based clusters may better capture immunological heterogeneity within exposure categories.

We next relate measured serological titers from Jian et al. [7] to our estimates of pseudo-escape that derive solely from variant frequency dynamics across countries. To assess whether pseudo-escape captures titer measurements, we compute serological titer distances as average log2 differences in titer values between pairs of variants. We compare these titer distances to distances in our pseudo-escape space (Fig. 6D), finding the distances between distinct variant pairs in the pseudo-escape space are correlated with these titer differences between variants ($R^{2}=0.402$). We bootstrap this analysis among 1,000 replicates to assess significance of this relationship (Fig. S17, p<0.001). Next, we subset by exposure history and find a similar relationship in most cohorts with the stronger correlations between serological titer distance and pseudo-immune escape in general being earlier exposures (WT, BA.5) and a weaker correlations observed in cohorts with later exposures (JN.1 and XBB) (Fig. S18).

We quantified which immune clusters are preferentially targeted by circulating lineages using the model-predicted pseudo-escape $\eta_{v}$. for each variant v to predict titers against that variant for individuals in each immune cluster and exposure history group (Fig. 6E–F). This relationship is delineated in Methods section ‘Regression of pseudo-escape onto neutralization titers’.

To assess variant-level escape against each cluster, we predict an escape burden as the negative of the predicted titer as a proxy for the escape potential against individuals of a certain background. Variants within JN.1 clade show elevated escape burden in specific clusters, with immune cluster 4 standing out as the most broadly targeted. This is consistent with the pseudo-escape patterns in Fig. 5F, where JN.1-family lineages exhibit high pseudo-escape along the dominant dimensions. Further, we find that immune cluster 4 shows a strong negative escape burden again viruses in clade 23B, likely owing the fact that individuals immune cluster 4 corresponds to a subset of individuals with past XBB infection (Fig. 6G). This is further supported by the fact that among all infection histories, pseudo-escape best predicts titers in individuals with past XBB infection including those with BA.5/BF.7 breakthrough infection (Fig. S19–S20).

In short, this shows that a sequence-only latent immunity model can recover an antigenic geometry that agrees with human serology and that learned pseudo-escape values can be used to predict cohort-level neutralization patterns. These properties make the latent representation useful for analyzing population susceptibility and explaining geographic variation in variant success, even when contemporaneous titer data are unavailable.

This approach can be applied to other antigenically variable pathogens, such as seasonal influenza, making it broadly applicable beyond SARS-CoV-2. In fact, there is more utility for pathogens with larger geographic differences in immunity since this approach enables to estimate the proportion of these latent immune pools in the population and how they vary geographically and over time alongside variant difference. By approximating antigenic differences using sequence data alone, this method offers for a deeper understanding of immune dynamics and how they shape variant success in the presence of immune escape. This enables an embedding similar to those from antigenic cartography but without the need for serological data and based purely on observed variant frequencies and estimated variant fitness.

## Discussion

Our study demonstrates the utility of multi-strain mechanistic models in interpreting variant frequency dynamics. This enables a more detailed picture of variant success in environments with heterogeneous population immunity. Our mechanistic grounding of variant fitness allows for investigations into trade-offs between intrinsic transmissibility increase and immune escape, prediction of epidemic dynamics from sequence data alone and inference of antigenic relatedness among variants from differences in success across geographies.

In particular, our latent factor model is most easily compared to the approaches of Meijers et al. [21] and Raharinirina et al. [22] that use cross-neutralization and deep mutational scanning data respectively to parameterize variant fitness. However, our approach differs significantly in that our model does not require any data other than sequence counts for each variant over time, enabling real-time analysis of fitness and heterogeneity in population immunity before cross-neutralization and deep mutational scanning data are available.

Despite these advances, there are limitations to our approach. Long-term forecasts remain difficult, particularly as new variants with unknown fitness profiles emerge. This framework suggests that considering both the escape against individual immune backgrounds and the diversity in human immune escape is most useful for improving forecasts of relative fitness. Our models, while powerful in estimating short-term variant dynamics, rely on assumptions about transmission mechanisms that may not always hold across different pathogens or contexts. In fact, as we’ve shown, it’s entirely possible for shifts in population immunity to change the dominant transmission mechanism.

Furthermore, the models considered here are deterministic in nature and do not explicitly model the emergence of variant viruses only the dynamics after their successful introduction. In reality, there are biological constraints on the types of variants that are produced in nature and even if there is a ‘true’ fitness boost, the chance for stochastic extinction of beneficial variants remains. These constraints present trouble for long-term forecasting as it will require a model of mutation or emergence, tying the potential for a variant to emerge with its potential to transmit in the current environment. Future work should focus on improving the integration of real-time genomic data with serological and epidemiological data, providing a more comprehensive understanding of variant dynamics over time.

In conclusion, our framework represents a significant advance in our understanding of viral evolution and transmission dynamics. By linking variant fitness to specific transmission mechanisms, we provide a more nuanced and accurate prediction of how variants will spread and impact population-level epidemic growth. The selective pressure metric and latent immunity model offer new tools for public health agencies to monitor viral evolution in real time, enabling proactive intervention and insight into the variant difference and wave potential. While our work has been applied to SARS-CoV-2, the methods developed here are broadly applicable to other evolving pathogens, offering a versatile approach for improving epidemic forecasting, variant monitoring, and overall pandemic preparedness.

## Materials and Methods

### Generating sequence counts

We prepared sequence count data sets using the Nextstrain-curated SARS-CoV-2 sequence metadata [33] which is created using the GISAID EpiCoV database [34]. These sequences were tallied according to either their annotated Nextstrain clade or Pango lineage [12] depending on the data set to produce sequence count for each variant, for each day over the period of interest, and in each country analyzed.

### Likelihood of sequence counts given frequencies

The models discussed in this paper use observed counts of variant sequences to inform the underlying variant frequency in the population. This is accomplished using a multinomial likelihood, so that given count of sequences $S_{v}(t)$ of variant v at time t and total sequences N(t) collected at time t, we have that

(12) $$S_{v}(t)\sim \text{Multinomial}\left(N(t),f_{v}(t)\right),$$

where $f_{v}(t)$ is the frequency of variant v at time t. This is a simple model of sequence counts to frequencies and does not account for over-dispersion of sequence counts relative to a multinomial. However, all models can be extended to estimate and account for over-dispersion by replacing the above likelihood with a Dirichlet-Multinomial likelihood.

### Approximate Gaussian processes for relative fitness estimation

To generate smooth non-parametric estimates of variant growth rates, we develop a Gaussian process based model for relative fitnesses. That is, we model the relative fitness for each variant over time $\lambda_{v}(t)$ as a multivariate normal distribution:

(13) $$\lambda_{v}\sim \text{Normal}(\mu ,\Sigma )$$

(14) $$\Sigma_{s,t}=K_{\theta }(s,t),$$

where $K_{\theta }$ is a potentially parameterized kernel function. This induces a structure on the covariance of the relative fitness values over time points s and t.

For computational efficiency, we implement a Hilbert Space Gaussian Process (HSGP) approximation instead of fitting V independent Gaussian processes. This approximation allows us to share basis functions between variants [28]. Under this approximation, the relative fitnesses are computed as

(15) $$\lambda_{v}\left(t\right)\approx \sum_{j=1}^{m}S_{\theta }{\left(\sqrt{\mu_{j}}\right)}^{\frac{1}{2}}\cdot \phi_{j}\left(t\right)\cdot \beta_{j},$$

where $S_{\theta }$ is the spectral density of the kernel $K_{\theta },\mu_{j}$ and $\phi_{j}$ are the eigenvalues and eigenfunctions of the Laplacian, and $\beta_{j}\sim \text{Normal}(0,1)$ [28]. Since the eigenvalues and eigenfunctions are shared across variants, this allows us to re-use values across variants, simplifying the computation to a matrix multiplication as

(16) $$\lambda_{t}=\Phi_{t}\sqrt{S_{\theta }}\beta .$$

For the analyses in this paper, we use this approximate Gaussian process with a Matérn 5/2 kernel and shared hyperparameters across variants. We demonstrate this model for simulated data from Fig. S1 and show resulting relative fitnesses through time in Fig. S2.

### Correlations are insufficient for mechanism identification

To assess how vaccination uptake affects the growth advantage of a variant with increased transmissibility, we simulate the spread of a more transmissible variant across populations with different initial past exposure and vaccination levels. This enables us to isolate the effects of transmissibility within different immunity landscapes, examining how relative fitness and growth advantage shift based on population vaccination coverage alone in the absence of immune escape. We begin with the 2-variant SIR model described in Supplementary Text S1. We simulate this model for 100 days with generation time τ=1/γ=3.0 days, $R_{0,W}=1.4,I_{W}(0)=100$ individuals, $I_{v}(0)=1$ individual, a 50% transmissibility increase ρ=0.5, and no immune escape η=0.0. We divide the period into early and late epidemic with the breakpoint being t=50. In Fig. 3D–E, we estimate the log growth advantage for the variant in the early and full periods using a logit-linear model

(17) $$\text{log}\left(\frac{f_{v}(t)}{1-f_{v}(t)}\right)=\beta t/\tau +\alpha ,$$

where we take the model slope β to be our log growth advantage.

We repeat these simulations for a range of vaccination levels starting from 0% and ending at 65%.

### Predicting epidemic growth rate from selective pressure

The derivation of the selective pressure metric shows that the selective pressure can be a useful tool in predicting the epidemic growth rate. To develop a predictive model of epidemic growth rate using selective pressure, we begin by generating estimates of selective pressure and epidemic growth rate from a period with high sequencing and case surveillance.

We take sequence count and case count data from all states in the United States between January 2021 and November 2022. State-level daily case counts were obtained from USAFacts downloaded on August 7, 2024 at https://usafacts.org/visualizations/coronavirus-covid-19-spread-map/.

Using the sequence counts, we compute selective pressure estimates from relative fitness and frequencies estimated with our approximate Gaussian process relative fitness model. From the case data, we derive the empirical growth rate using a 14-day moving average on case counts ${\overset{ˆ}{C}}_{t}$ and computing the empirical growth rate as ${\overset{ˆ}{r}}_{t}=\text{log}\left({\overset{ˆ}{C}}_{t}\right)-\text{log}\left({\overset{ˆ}{C}}_{t-1}\right)$. We then use the past 28 days of selective pressure to predict the empirical growth rate.

We use a gradient boosting regressor model which is fit using a mean absolute error loss function. This model was selected as it achieved the minimal error via time series cross-validation averaged across 10 splits among candidate models (Fig. S10). The candidate models include linear regression, ridge regression, Lasso regression, random forests, and gradient-boosted trees as implemented in scikit-learn [35]. We additionally tune the hyperparameters of this model using grid search cross-validation.

We validate our model by comparing our predicted epidemic growth rates to held-out case data for US states, South Africa, South Korea, the United Kingdom, and additionally to estimates of the epidemic growth rates in England derived from data from the Office for National Statistics (ONS) Coronavirus Infection Survey [32]. Estimates of prevalence from the ONS Infection Survey were obtained for January 2022 to September 2022 from www.ons.gov.uk/peoplepopulationandcommunity/healthandsocialcare/conditionsanddiseases/datasets/coronaviruscovid19infectionsurveydata Epidemic growth rates are computed on this data in the same way as the state-level analysis.

### Latent immune factor model

We show that relative fitness dynamics can be explained by low-dimensional immunity when transmission dynamics are described with compartmental models (Supplementary Text S1). This motivates a model to learn this low-dimensional structure that is inspired by latent-factor models. We start by assuming that the relative fitness of variant v at time t and in geographic location g can be described by D latent factors so that

(18) $$\lambda_{v}^{g}\left(t\right)=\sum_{d=1}^{D}\eta_{v,d}\phi_{d}^{g}\left(t\right).$$

As the structure here resembles Equation 43, we call $\eta_{v,d}$ “pseudo-escape” of variant v from group d and $\phi_{d}^{g}$ “pseudo-immunity” group d in geographic location g. To make this more consistent with our intuition here, we model $\phi_{d}^{g}$ to be in [0, 1] and model it as smoothly varying in time. We model $\text{logit}\left(\phi_{d}^{g}\right)$ using 4th order splines with 6 knots placed uniformly over the time period modeled. Though we choose to model these latent factors with splines, other models would work here. For example, one alternative would be the approximate Gaussian processes described above. Additionally, in order to ensure identifiability of the parameter estimates, we fix some base variant $v^{*}$ which fitness is defined relative to, so that $\eta_{v^{*},d}=0$ for all 1≤d≤D. For the same reason, we fix the order of components, so that the components are numbered in decreasing order by their share in the arbitrarily defined base geography.

We apply this model to SARS-CoV-2 sequence counts in the period between March 2023 to March 2024 for 14 countries. To access the necessary number of immune dimensions, we vary the number of immune dimensions between D=2 to D=12. Looking at the loss for the latent factor model for increasing D, we choose D=8 for our primary analysis by noting the point at which the loss function seems to stagnate with increasing D i.e. the “elbow” method (Fig. S11).

We compare the distances between variant pairs in our estimated pseudo-escape space to distances in log2 titer. Using human titer data from Jian et al [7], we compute neutralization titer distances as the average of differences in log2 neutralization titers between pairs of variants for a cohort of individuals. This analysis is repeated among 1,000 bootstrapped samples to create a distribution of $R^{2}$ values (Fig. S17). Additionally, we subset this by exposure history and repeat this analysis to find which exposure groups best explain distances in pseudo-escape space (Fig. S18).

### Regression of pseudo-escape onto neutralization titers

To assess the relationship between our estimated pseudo-escape values and empirical neutralization titers, we use human titer data from Jian et al. [7]. For each exposure group, we have a set of neutralization titers measured against multiple variants. Our goal is to determine whether variation in titers across variants can be explained by the pseudo-escape values inferred from our latent immune factor model.

Let $t_{i,v}$ denote the neutralization titer of individual i against variant v. For each exposure group G, we first aggregate titers by computing the group-level mean and applying a log_2_ transformation with a pseudocount of one, i.e.


(19) $$y_{v}^{G}={\text{log}}_{2}\left({\text{mean}}_{i\in G}\left(t_{i,v}\right)+1\right).$$

Let $\eta_{v}\in R^{D}$ denote the pseudo-escape vector of variant v estimated from the latent immune factor model across D latent factors. We then fit an ordinary least squares (OLS) model separately for each exposure group,

(20) $$y_{v}^{G}=\beta_{0}+\beta^{⊤}\eta_{v}+\varepsilon_{g,v},$$

where $\beta_{0}$ is an intercept term and β is a vector of regression coefficients mapping latent factors to predicted group-level titers. This yields a coefficient of determination $R_{g}^{2}$ for each group, quantifying how well variation in pseudo-escape explains variation in group-level titers.

To assess statistical significance, we performed a permutation test in which the association between variants and their pseudo-escape vectors was randomly permuted 1,000 times. For each permutation, we re-fit the OLS model and recorded the resulting $R_{g}^{2}$, yielding a null distribution under the hypothesis that pseudo-escape and titers are unrelated. The empirical p-value for each group was computed as the fraction of permutations with $R_{g}^{2}$ greater than or equal to the observed $R_{g}^{2}$. This procedure controls for the correlation structure in the titer data while directly testing whether the inferred pseudo-escape values have predictive power for neutralization measurements.

We summarize the results across groups by reporting both the observed $R^{2}$ values and the permutation-derived p-values (Fig. S19 and S20). This analysis identifies exposure groups where pseudo-escape provides a statistically significant explanation of the neutralization response profile.

## Supplementary Material

1

## Data Availability

Source code used to generate figures, model implementations, and sequence count data are available at github.com/blab/relative-fitness-mechanisms.
