# Supplementary material for: Frequency dynamics predict viral fitness, antigenic relationships and epidemic growth
Source: medRxiv. 2025 Jan 23:2024.12.02.24318334. Originally published 2024 Dec 3. Preprint. [Version 2] doi: 10.1101/2024.12.02.24318334 (PMC11643185; doi:10.1101/2024.12.02.24318334)
Supplement: Supplementary file 1 [file NIHPP2024.12.02.24318334V2-supplement-1.pdf]

medRxiv preprint doi: https://doi.org/10.1101/2024.12.02.24318334; this version posted January 23, 2025. The copyright holder for this preprint (which was not certified by peer review) is the author/funder, who has granted medRxiv a license to display the preprint in perpetuity.
It is made available under a CC-BY 4.0 International license .

Supplementary Information for
Frequency dynamics predict viral fitness, antigenic relationships and epidemic growth

Supplementary Text

S1 Exponentially growing populations to frequency dynamics

We consider a viral population consisting of V exponentially-growing variant viruses each with prevalence I_v. Defining the time-varying growth rate for the prevalence of variant v as $r_v(t)$, we can model the prevalence using an ordinary differential equation

$$\frac{dI_v}{dt} = r_v(t)I_v(t), \quad v = 1, 2, \ldots, V. \tag{19}$$

The above differential equation has a known solution in terms of the integral of the time-varying growth rate and initial prevalence,

$$I_v(t) = I_v(0) \exp\left(\int_0^t r_v(s)ds\right), \tag{20}$$

where $I_v(0)$ is the initial prevalence of variant v.

Now turning to the frequency dynamics of the population, we write the frequency of variant v in the population as $f_v(t) = I_v(t)/\sum_{u=1}^{V} I_u(t)$. This allows us to derive an ODE for variant frequency in terms of the variant growth rates using the quotient rule for differentiation

$$\frac{df_v}{dt} = f_v\left(\sum_{u=1}^{V}[r_v(t) - r_u(t)]f_u\right) \tag{21}$$

$$= f_v\left(r_v(t) - \sum_{u=1}^{V} r_u(t)f_u\right). \tag{22}$$

This system of differential equations resembles a logistic growth equation and can be shown to have the following solution in terms of the initial frequencies $f_v(0)$ and the variant growth rates

$$f_v(t) = \frac{f_v(0)\exp(\int_0^t r_v(s)ds)}{\sum_{u=1}^{V} f_u(0)\exp(\int_0^t r_u(s)ds)}. \tag{23}$$

The above representation of the variant frequency will serve as a centerpiece for many of the arguments to follow. We see that by tracking the rate at which variant viruses are spreading, we can construct the corresponding frequency dynamics without knowing the absolute prevalence of any variant.

medRxiv preprint doi: https://doi.org/10.1101/2024.12.02.24318334; this version posted January 23, 2025. The copyright holder for this preprint (which was not certified by peer review) is the author/funder, who has granted medRxiv a license to display the preprint in perpetuity.
It is made available under a CC-BY 4.0 International license .

Relative frequency and relative fitness Using the above equation for the variant frequencies, we can write the relative frequency of variant v over u as $x_{v,u}(t) = f_v(t)/f_u(t)$ to see

$$x_{v,u}(t) = \frac{f_v(t)}{f_u(t)} = \frac{f_v(0)}{f_u(0)} \exp\left(\int_0^t [r_v(s) - r_u(s)]ds\right) \tag{24}$$

$$= x_{v,u}(0) \exp\left(\int_0^t \lambda_{v,u}(s)ds\right). \tag{25}$$

Notice this relative frequency change depends on the initial relative frequencies and the *relative fitness* $\lambda_{v,u}(t) = r_v(t) - r_u(t)$ of v over u. This relative fitness has the same units as the exponential growth rate (e.g. per day). Using the definition of relative fitness, we can notice that

$$\lambda_{v,u}(t) = r_v(t) - r_u(t) = \frac{d}{dt}\left[\log\left(x_{v,u}(t)\right)\right] = -\lambda_{u,v}(t). \tag{26}$$

We can see that there is a symmetry in the relative fitnesses and that the associated frequency dynamics depend on the differences between relative fitnesses. This suggests that absolute fitness (in terms of the growth of infections) may not be inferable from frequencies alone. This definition of relative fitness becomes essential in describing various existing modeling approaches for frequency dynamic data and motivates possible extensions since we can represent these models as having the form:

$$f_v(t) = \frac{f_v(0)\exp(\int_0^t \lambda_{v,v^*}(s)ds)}{\sum_{u=1}^V f_u(0)\exp(\int_0^t \lambda_{u,v^*}(s)ds)}, \tag{27}$$

where the growth rate of v is expressed as relative to an arbitrary pivot variant v^*.

Cumulative relative-fitness and frequency change Above we saw that within our framework frequency change over time intervals depends only on the cumulative relative fitness over time intervals $\Lambda_{v,u}(0,t) = \int_0^t \lambda_{v,u}(s)ds$. We can then characterize approaches for modeling frequency change in terms of how they represent, estimate, and forecast these relative fitnesses. This framework includes various existing methods for analyzing frequency data such as the seasonal influenza forecasting models of Lässig and Łuksza [34] and Huddleston et al [35], multinomial logistic regression for frequency estimation [13] and the SARS-CoV-2 mutational fitness model of Obermeyer et al [36].

Though this framework can be used to describe existing statistical methods for frequency modeling, it is also applicable to traditional compartmental models of epidemics. In fact, applying these ideas to compartmental models enables to see how mechanistic assumptions on the transmission process determine relative fitness of variant viruses.

Two-strain SIR For simplicity, we will begin by analyzing a two-strain SIR model in which a variant virus v can differ from wildtype virus wt by increased intrinsic transmissibility (via η_T) and immune escape against wild-type immunity (via η_E). This gives a system of 5 ordinary differential equations

medRxiv preprint doi: https://doi.org/10.1101/2024.12.02.24318334; this version posted January 23, 2025. The copyright holder for this preprint (which was not certified by peer review) is the author/funder, who has granted medRxiv a license to display the preprint in perpetuity.
It is made available under a CC-BY 4.0 International license .

$$\frac{dS}{dt} = -\beta S I_W - \beta\rho S I_v, \tag{28}$$
$$\frac{dI_W}{dt} = \beta S I_W - \gamma I_W, \tag{29}$$
$$\frac{dI_v}{dt} = \beta(1+\rho) S I_v + \beta(1+\rho)\eta\phi_W I_v - \gamma I_v, \tag{30}$$
$$\frac{d\phi_W}{dt} = \gamma I_W - \beta(1+\rho)\eta\phi_W I_v, \tag{31}$$
$$\frac{d\phi_v}{dt} = \gamma I_v, \tag{32}$$

where I_W denotes wild-type prevalence, I_v denotes variant prevalence and ϕ_W denotes immunity derived from wild-type infection. In this model, the variant virus can infect both susceptible individuals S and individuals with immunity to wild-type virus ϕ_W. Increased intrinsic transmissibility increases the baseline transmission rate from β in wild-type to $\beta\rho$ in the variant virus and immune escape increases the transmission rate against those with wildtype immunity, so that the at-risk population is $\eta\phi_W$.

Writing that $r_W(t) = \beta S - \gamma$ and $r_v(t) = \rho\beta S + \beta(1+\rho)\eta\phi_W - \gamma$, we can then write the relative fitnesses as:

$$\lambda_{v,W}(t) = \rho\beta S(t) + (1+\rho)\eta\beta\phi_W(t). \tag{33}$$

From this representation of relative fitness, we can see that given fixed increases to overall transmission ($\rho > 0$) or immune escape ($\eta > 0$), the observed fitness boost at the level of variant relative fitness still depends on the proportion of the population at risk for infection.

n-strain SIR This model can also be extended to an n-strain SIR model where each variant strain v_i with $2 \leq i \leq n$ is described by its own advantage parameters $\theta_i = (\rho^{(i)}, \eta^{(i)})$ relative to the wildtype ($\theta_W = \theta_1 = (0, 0)$)

$$\frac{dS}{dt} = -\beta S I_W - \beta(1+\rho) S I_{v_i} \tag{34}$$
$$\frac{dI_W}{dt} = \beta S I_W - \gamma I_W \tag{35}$$
$$\frac{dI_{v_i}}{dt} = \beta(1+\rho_i) S I_{v_i} + \beta(1+\rho_i)\eta_i\phi_W I_{v_i} - \gamma I_{v_i} \tag{36}$$
$$\frac{d\phi_W}{dt} = \gamma I_W - \beta(1+\rho_i)\eta_i\phi_W I_{v_i} \tag{37}$$
$$\frac{d\phi_{v_i}}{dt} = \gamma I_{v_i}, \quad i \in \{2, \ldots, n\}. \tag{38}$$

In this formulation, the variant viruses compete only for susceptible population and those with previous wild-type infection. This formulation can be generalized to allow for competition between all variants for any exposure history and will be discussed in the following sections. In Fig. 1, we implement and simulate a 3-strain model with wildtype as above, an escape variant E with $\theta_2 = (0, \eta)$, and a transmissibility increase variant T with $\theta_3 = (\rho, 0)$.

medRxiv preprint doi: https://doi.org/10.1101/2024.12.02.24318334; this version posted January 23, 2025. The copyright holder for this preprint (which was not certified by peer review) is the author/funder, who has granted medRxiv a license to display the preprint in perpetuity.
It is made available under a CC-BY 4.0 International license .

Models of immune escape against heterogeneous backgrounds We'll now consider a model where all hosts are assumed to fall into one of B immune backgrounds ϕ_b for $b = 1, \dots, B$. We assume that infection by each variant v then leaves recovered hosts in the corresponding immune background of the most recent infection b_v. Variant transmission then occurs via immune escape against a background leading to a matrix of escape rates $\boldsymbol{\eta} = \eta_{v,b}$ for variants v and background b.

We can then write the system of ordinary differential equations as

$$\frac{dI_v}{dt} = \beta \sum_{1 \leq b \leq B} \eta_{v,b} \phi_b I_v - \gamma I_v, \quad v = 1, \dots, V \tag{39}$$

$$\frac{d\phi_b}{dt} = -\beta \sum_{1 \leq v \leq V} \eta_{v,b} \phi_b I_v + \sum_{v:\ b_v = b} \gamma I_v. \tag{40}$$

With this model, susceptible and recovered compartments in the standard SIR model can be thought of as immune backgrounds. This allows us to represent the standard SIR model as $S = \phi_S$, $I = I_W$, $R = \phi_W$ and $\eta_{W,S} = 1, \eta_{W,W} = 0$ and $b_W = W$. We can also think of the two-strain SIR with $\rho = 1$ as a special case of this model where we set $S = \phi_S, \eta_{W,S} = 1, \eta_{W,W} = 0, \eta_{v,S} = 1, \eta_{v,W} = \eta$ and keep all other parameters the same.

With this formulation of immune escape, we can then write the relative fitnesses in terms of the escape rates $\eta_{v,b}$ and the immune background proportions ϕ_b as

$$\lambda_{v,u}(t) = \beta \sum_{1 \leq b \leq B} (\eta_{v,b} - \eta_{u,b}) \phi_b(t). \tag{41}$$

Under this model of immune escape, we can see relative fitness among variants can be decomposed into differences in immune escape among immune backgrounds within a population. Due to the dependence here on the proportion of each immune background in determining fitness, this suggests that the overall distribution of susceptibility to strains is potentially an important consideration when translating individual-level measures of immune escape to population-level estimates of variant fitness. Understanding the size and complexity of this immune space may therefore be useful for parameterization and forecasting of variant frequencies. However, the extent to which modeling this complexity affects estimates of relative fitness also depends on how quickly the distribution of immune backgrounds change i.e. $\frac{d\phi_b}{dt}$.

Though the derivation above uses a simplified model of using most recent infection to sort individuals into an immune group, we show that a more complicated model that accounts for the entire exposure history of the host also gives a similar decomposition to relative fitness in Supplementary Text S3.

S2 Revisiting existing models for frequency growth

Using the theory developed for exponentially-growing variant populations, we now re-visit existing methods for modeling viral frequency dynamics.

medRxiv preprint doi: https://doi.org/10.1101/2024.12.02.24318334; this version posted January 23, 2025. The copyright holder for this preprint (which was not certified by peer review) is the author/funder, who has granted medRxiv a license to display the preprint in perpetuity.
It is made available under a CC-BY 4.0 International license .

Multinomial Logistic Regression We begin with multinomial logistic regression (MLR) with fixed relative fitness. This model can be written as

$$f_v(t) = \frac{f_v(0)\exp(\lambda_v t)}{\sum_u f_u(0)\exp(\lambda_u t)}, \tag{42}$$

where $f_v(t)$ is the frequency of variant v at time t and λ_v is the relative fitness of variant v. This provides estimates of the relative fitness compared to some reference strain u^* for which $\lambda_{u^*} = 0$. In this model, initial frequencies $f_v(0)$ and relative fitness λ_v are estimated from frequency dynamics. Converting this estimate to an estimate of transmission advantage (relative effective reproduction number) requires assuming a delta distribution of the generation time [37].

Comparing this to equation 33, we can see this model of fixed relative fitness results from assuming that the at-risk populations are constant over-time. This assumption is useful since it requires no outside knowledge of the at-risk population and relative infection rates, though this may be less useful for longer forecasts or when there is large turnover in at-risk populations due to infection.

Fitness models of seasonal influenza Motivated by the observed antigenic evolution of seasonal influenza, Lässig and Łuksza [34] and Huddleston et al [35] approximate the cumulative relative fitness between influenza seasons on the level of individual strains as

$$\Lambda_{v,u}(t+\Delta t, t) = (\beta_1 x_{v,1} + \cdots + \beta_p x_{v,p})\Delta t = (\boldsymbol{\beta}\cdot\boldsymbol{x}_v)\Delta t, \tag{43}$$

where the relative fitness is determined by strain-specific predictors $\boldsymbol{x}_v$ and the regression parameter $\boldsymbol{\beta}_v$ are estimated.

This formulation fits neatly into the framework we've developed as the cumulative fitness here can be written as the integral of a relative fitness $\lambda_{v,u} = \boldsymbol{\beta}\cdot\boldsymbol{x}_v$ over the time period of interest:

$$\Lambda_{v,u}(t+\Delta t, t) = \int_t^{t+\Delta t} \lambda_{v,u}(s)ds \; = \int_t^{t+\Delta t} (\boldsymbol{\beta}\cdot\boldsymbol{x}_v)ds. \tag{44}$$

Therefore, these models can be thought as regression-based predictors of relative fitness where frequency and external covariates contribute to estimated relative fitness.

S3 Relative fitness for full immune history models

We show that the simple background model is consistent with an expanded immune history model. Beginning with the model from Lazebnik and Bunimovich-Mendrazitsky 2022 [38], we consider the differential equation for the individuals with strain infection history J and current infecting strain i $R_J I_i$

$$\frac{dR_J I_i}{dt} = -\gamma_{J,i} R_J I_i + \beta_{J,i} R_J \sum_{K\in P(M), i\notin K} R_K I_i. \tag{45}$$

medRxiv preprint doi: https://doi.org/10.1101/2024.12.02.24318334; this version posted January 23, 2025. The copyright holder for this preprint (which was not certified by peer review) is the author/funder, who has granted medRxiv a license to display the preprint in perpetuity.
It is made available under a CC-BY 4.0 International license .

Here, infection can occur from any individual infected with strain i assuming their past immune history does not include i and the infected are any recovered individual with immune history J R_J. To compute the strain growth rate, we can sum over all possible immune histories for individuals infected with strain i, so that

$$\frac{dI_i}{dt} = \sum_{J \in P(M), i \notin J} \frac{dR_J I_i}{dt} \tag{46}$$

$$= -\gamma_i I_i + \sum_{J \in P(M), i \notin J} \beta_{i,J} R_J \sum_{K \in P(M), i \notin K} R_K I_i \tag{47}$$

$$= -\gamma_i I_i + \sum_{J \in P(M), i \notin J} \beta_{i,J} R_J I_i \tag{48}$$

$$= \left(-\gamma_i + \sum_{J \in P(M), i \notin J} \beta_{i,J} R_J \right) I_i \tag{49}$$

$$= \left(-\gamma + \beta \sum_{J \in P(M), i \notin J} \eta_{i,J} R_J \right) I_i. \tag{50}$$

$$\tag{51}$$

Assuming that the transmission rate can be decomposed as a base transmission rate β and a strain i and immune history J specific escape rate $\eta_{i,J}$ and that the recovery rate is constant, we notice this is identical to our previous immune background model. Therefore, our relative fitnesses simplify to

$$\lambda_{i,j} = \beta \sum_{B \in P(M)} (\eta_{i,B} - \eta_{j,B}) R_B, \tag{52}$$

where for simplicity we define $\eta_{v,B} = 0$ if $v \in B$.

S4 Selective pressure and contribution to epidemic growth rates

In this section, we derive our selective pressure metric $\psi(t)$ and show how it contributes to the overall epidemic growth rate in the population.

Beginning again from our assumption of inhomogeneous exponential growth, we can write a differential equation for the total prevalence $I(t) = \sum_v I_v(t)$,

$$\frac{dI}{dt} = \sum_v \frac{dI_v}{dt} = \sum_v r_v(t) I_v(t) \tag{53}$$

$$= \left(\sum_v r_v(t) f_v(t) \right) I(t), \tag{54}$$

medRxiv preprint doi: https://doi.org/10.1101/2024.12.02.24318334; this version posted January 23, 2025. The copyright holder for this preprint (which was not certified by peer review) is the author/funder, who has granted medRxiv a license to display the preprint in perpetuity.
It is made available under a CC-BY 4.0 International license .

where we've used that $I_v(t) = f_v(t)I(t)$. This allows us to see that $\overline{r}(t) = \sum_v r_v(t)f_v(t)$ is the average growth rate of the prevalence. Re-writing the average in terms of some base exponential growth rate and the relative fitnesses so that $r_v(t) = \lambda_v(t) + r_W(t)$, we get that $\overline{r}(t) = \sum_v \lambda_v(t)f_v(t) + r_W(t)$. We can simplify this by writing $\overline{r}(t) = \overline{\lambda}(t) + r_W(t)$ where $\overline{\lambda}(t) = \sum_v \lambda_v(t)f_v(t)$ is the mean fitness of the population. We can now look at the rate of change in the average growth rate by taking its derivative

$$\begin{aligned}
\frac{d\overline{r}}{dt} &= \frac{dr_W}{dt} + \frac{d\overline{\lambda}}{dt} && (55)\\
&= \frac{dr_W}{dt} + \sum_v \left[\frac{d\lambda_v}{dt} f_v(t) + \lambda_v(t)\frac{df_v}{dt}\right] && (56)\\
&= \frac{dr_W}{dt} + \sum_v \left[\frac{d\lambda_v}{dt} f_v + \lambda_v f_v(\lambda_v - \overline{\lambda})\right] && (57)\\
&= \frac{dr_W}{dt} + \sum_v \frac{d\lambda_v}{dt} f_v(t) + \sum_v \lambda_v(\lambda_v - \overline{\lambda}) f_v && (58)\\
&= \frac{dr_W}{dt} + \sum_v \frac{d\lambda_v}{dt} f_v(t) + \sum_v \lambda_v^2 f_v - \overline{\lambda}\sum_v \lambda_v f_v && (59)\\
&= \frac{dr_W}{dt} + \mathbb{E}_{f(t)}\left[\frac{d\lambda_v}{dt}\right] + \mathbb{V}_{f(t)}[\lambda_v]. && (60)
\end{aligned}$$

Here, we've written the last line in terms of expectations relative to sampling according to the frequency distribution. This shows us that the change in the average growth rate of the epidemic can be written in terms of the growth rate of the pivot category, the mean rate of change in the relative fitness, and the variance of the relative fitnesses. We will call terms which can be computed in terms of quantities derived from frequencies alone the selective pressure

$$\psi(t) = \mathbb{E}_{f(t)}\left[\frac{d\lambda_v}{dt}\right] + \mathbb{V}_{f(t)}[\lambda_v]. \tag{61}$$

We can use this idea to directly write the prevalence in terms of the selective pressure and the base growth rate. First, we define a cumulative selective pressure

$$\Psi(t) = \int_0^t \psi(s)ds. \tag{62}$$

We can then use this to reconstruct the relative incidence

$$\begin{aligned}
\frac{I(t)}{I(0)} &= \exp\left(\int_0^t \overline{r}(s)ds\right) && (63)\\
&= \exp\left(\int_0^t [r_W(s) + \Psi(s)]ds\right). && (64)
\end{aligned}$$

medRxiv preprint doi: https://doi.org/10.1101/2024.12.02.24318334; this version posted January 23, 2025. The copyright holder for this preprint (which was not certified by peer review) is the author/funder, who has granted medRxiv a license to display the preprint in perpetuity.
It is made available under a CC-BY 4.0 International license .

Supplementary Figures

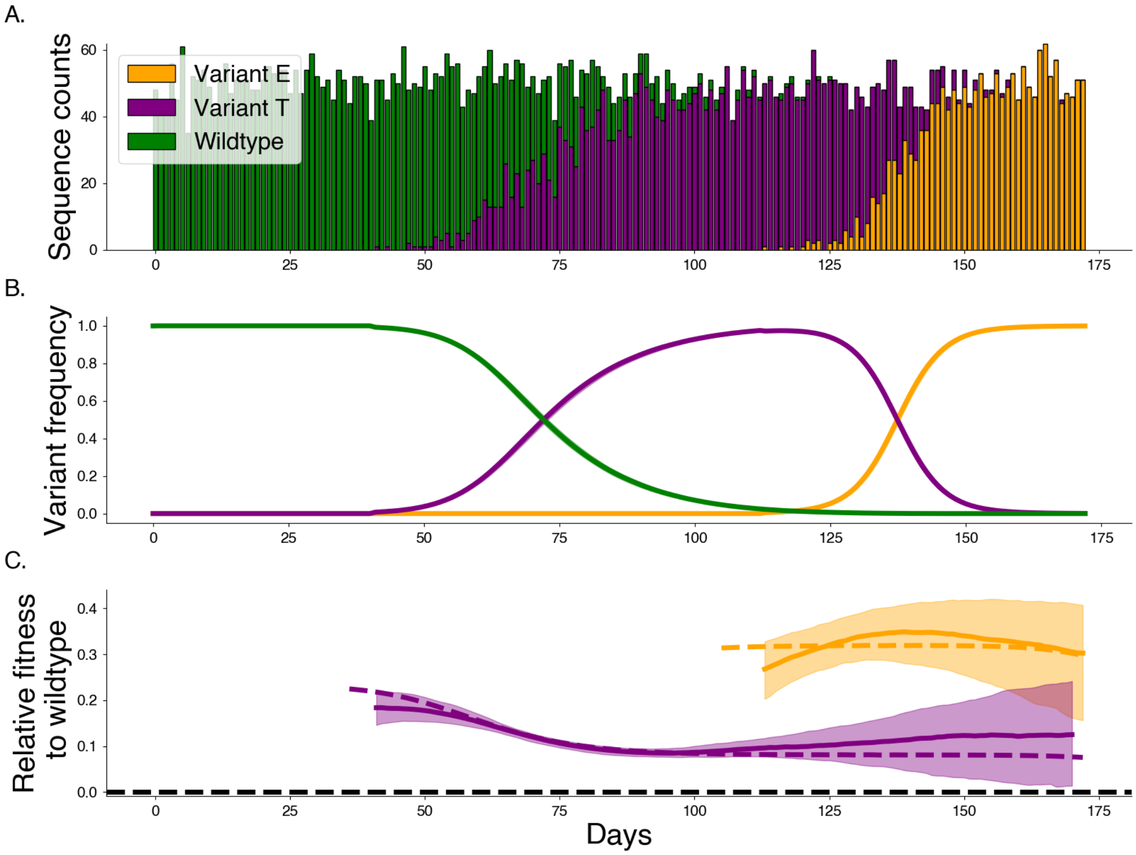

Fig. S1. Estimating relative fitness with Gaussian processes. Gaussian processes allow us a non-parametric estimate of the relative fitness for variants through time. This figure uses Gaussian processes to model the 3 variant example shown in Fig. 1. A. Synthetic sequence counts generated using a multinomial distribution with frequencies from Fig. 1C. B. Frequencies and posterior frequencies according to Gaussian process model. Intervals show the 80% credible interval. C. Relative fitnesses. Dashed line shows true relative fitnesses from underlying mechanistic model.

medRxiv preprint doi: https://doi.org/10.1101/2024.12.02.24318334; this version posted January 23, 2025. The copyright holder for this preprint (which was not certified by peer review) is the author/funder, who has granted medRxiv a license to display the preprint in perpetuity.
It is made available under a CC-BY 4.0 International license .

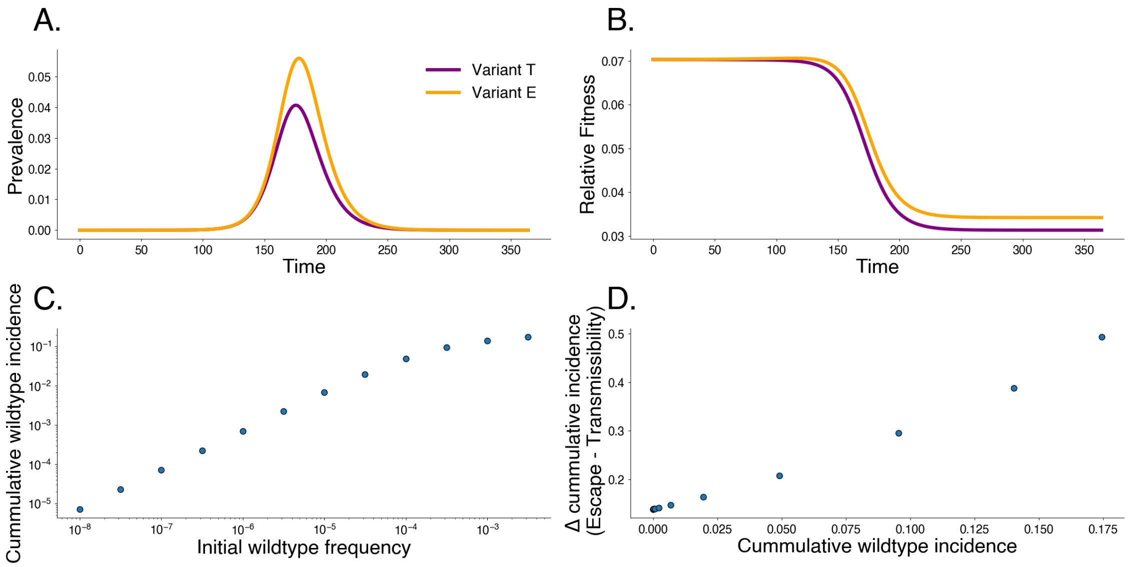

Fig. S2. Differences in fitness mechanisms impact frequency and prevalence in the short-term. Comparing simulations from two independent two-variant systems with either an escape variant E (orange) or a transmissibility variant T (purple). We fix the initial relative fitness for the two variants using Equation 4 and simulate dynamics for 365 days. A. The prevalence for the variants. B. The relative fitness from the variants. C. The cumulative wildtype incidence as a function of the initial wildtype frequency. D. The difference between the cumulative incidence between the escape variant and the transmissibility variant as a function of wildtype incidence.

medRxiv preprint doi: https://doi.org/10.1101/2024.12.02.24318334; this version posted January 23, 2025. The copyright holder for this preprint (which was not certified by peer review) is the author/funder, who has granted medRxiv a license to display the preprint in perpetuity.
It is made available under a CC-BY 4.0 International license .

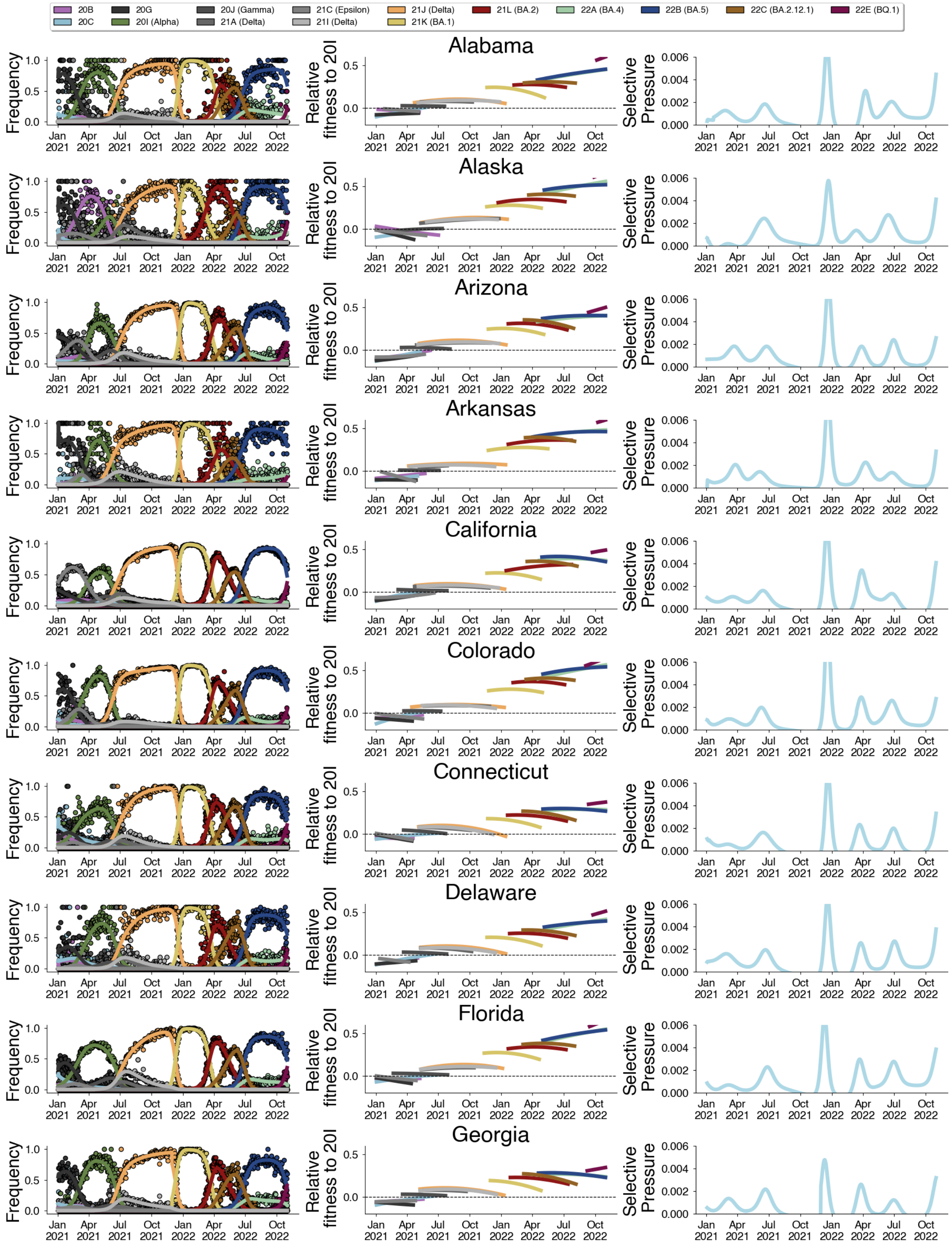

Fig. S3. Estimated variant frequencies, relative fitnesses, and selective pressure. Alabama through Georgia.

medRxiv preprint doi: https://doi.org/10.1101/2024.12.02.24318334; this version posted January 23, 2025. The copyright holder for this preprint (which was not certified by peer review) is the author/funder, who has granted medRxiv a license to display the preprint in perpetuity.
It is made available under a CC-BY 4.0 International license .

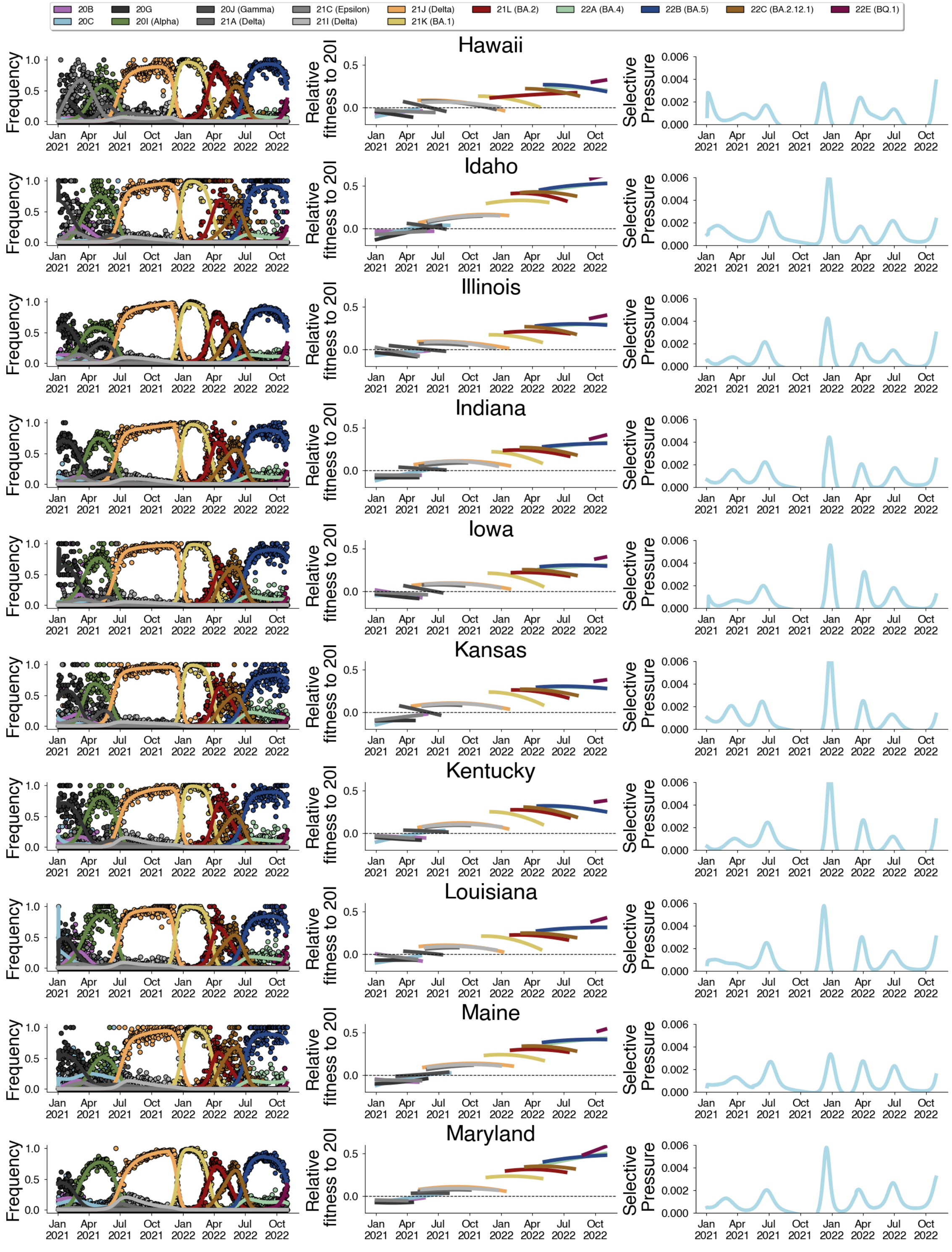

Fig. S4. Estimated variant frequencies, relative fitnesses, and selective pressure. Hawaii to Maryland.

medRxiv preprint doi: https://doi.org/10.1101/2024.12.02.24318334; this version posted January 23, 2025. The copyright holder for this preprint
(which was not certified by peer review) is the author/funder, who has granted medRxiv a license to display the preprint in perpetuity.
It is made available under a CC-BY 4.0 International license .

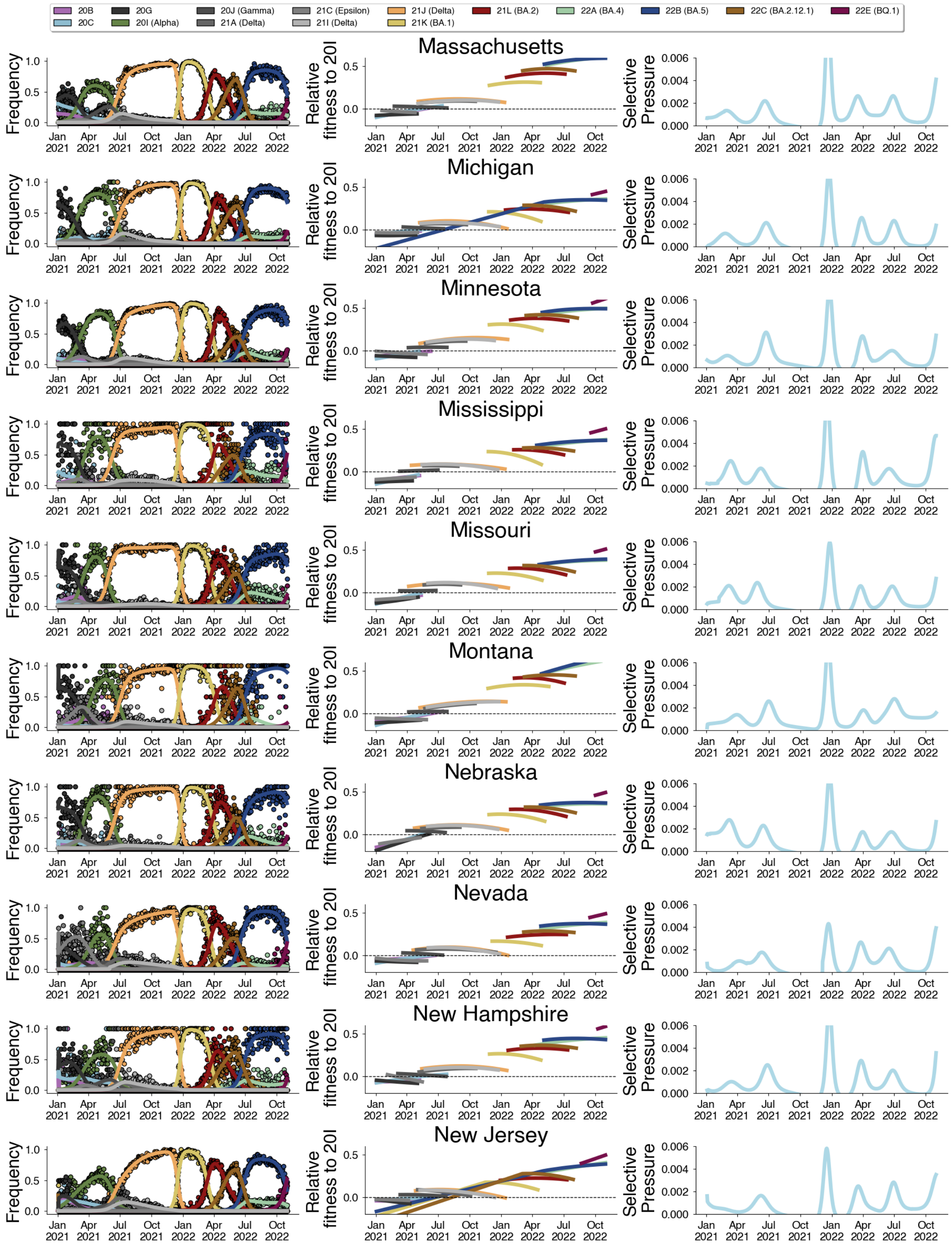

Fig. S5. Estimated variant frequencies, relative fitnesses, and selective pressure. Massachusetts to New Jersey.

medRxiv preprint doi: https://doi.org/10.1101/2024.12.02.24318334; this version posted January 23, 2025. The copyright holder for this preprint (which was not certified by peer review) is the author/funder, who has granted medRxiv a license to display the preprint in perpetuity.
It is made available under a CC-BY 4.0 International license .

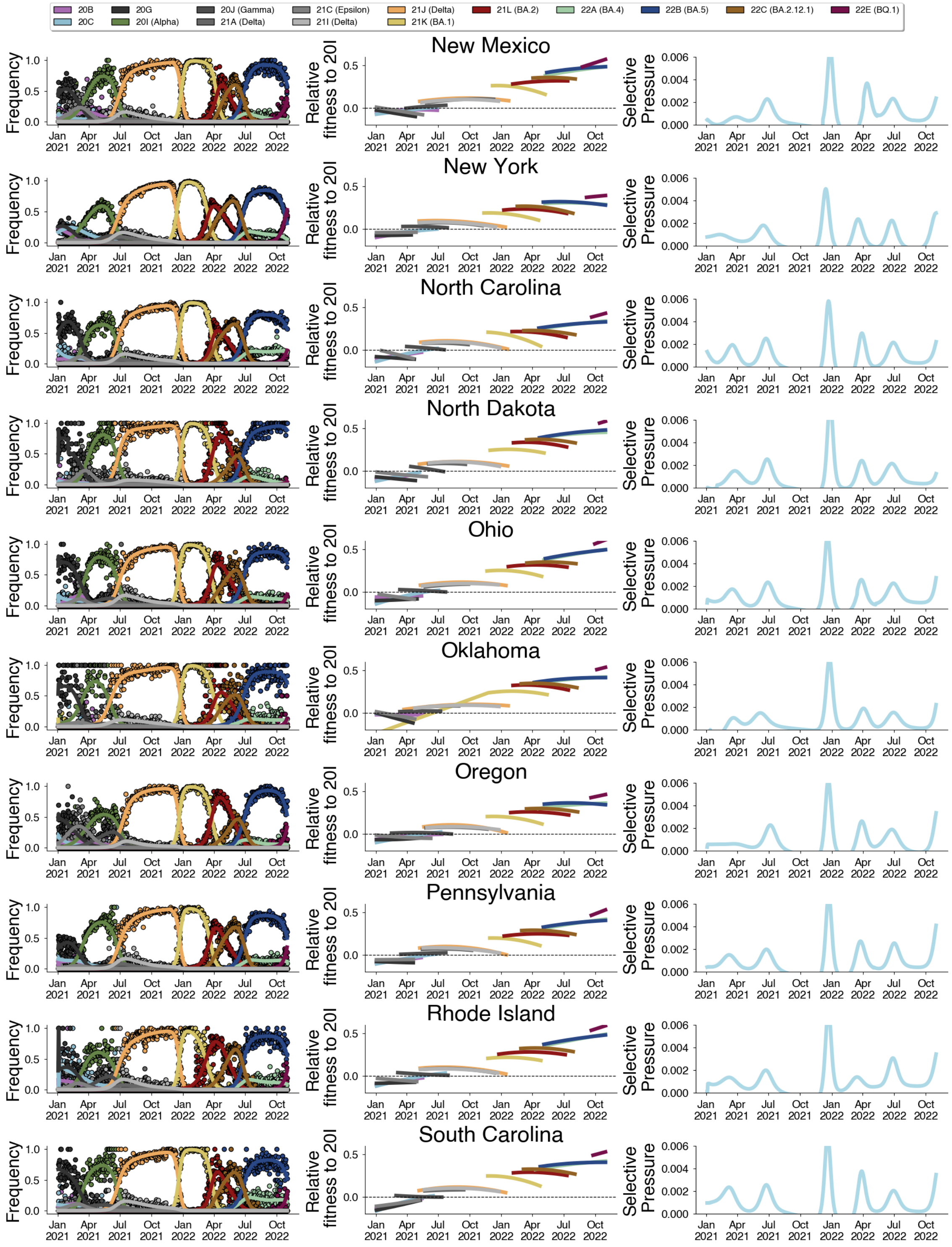

Fig. S6. Estimated variant frequencies, relative fitnesses, and selective pressure. New Mexico to South Carolina.

medRxiv preprint doi: https://doi.org/10.1101/2024.12.02.24318334; this version posted January 23, 2025. The copyright holder for this preprint (which was not certified by peer review) is the author/funder, who has granted medRxiv a license to display the preprint in perpetuity.
It is made available under a CC-BY 4.0 International license .

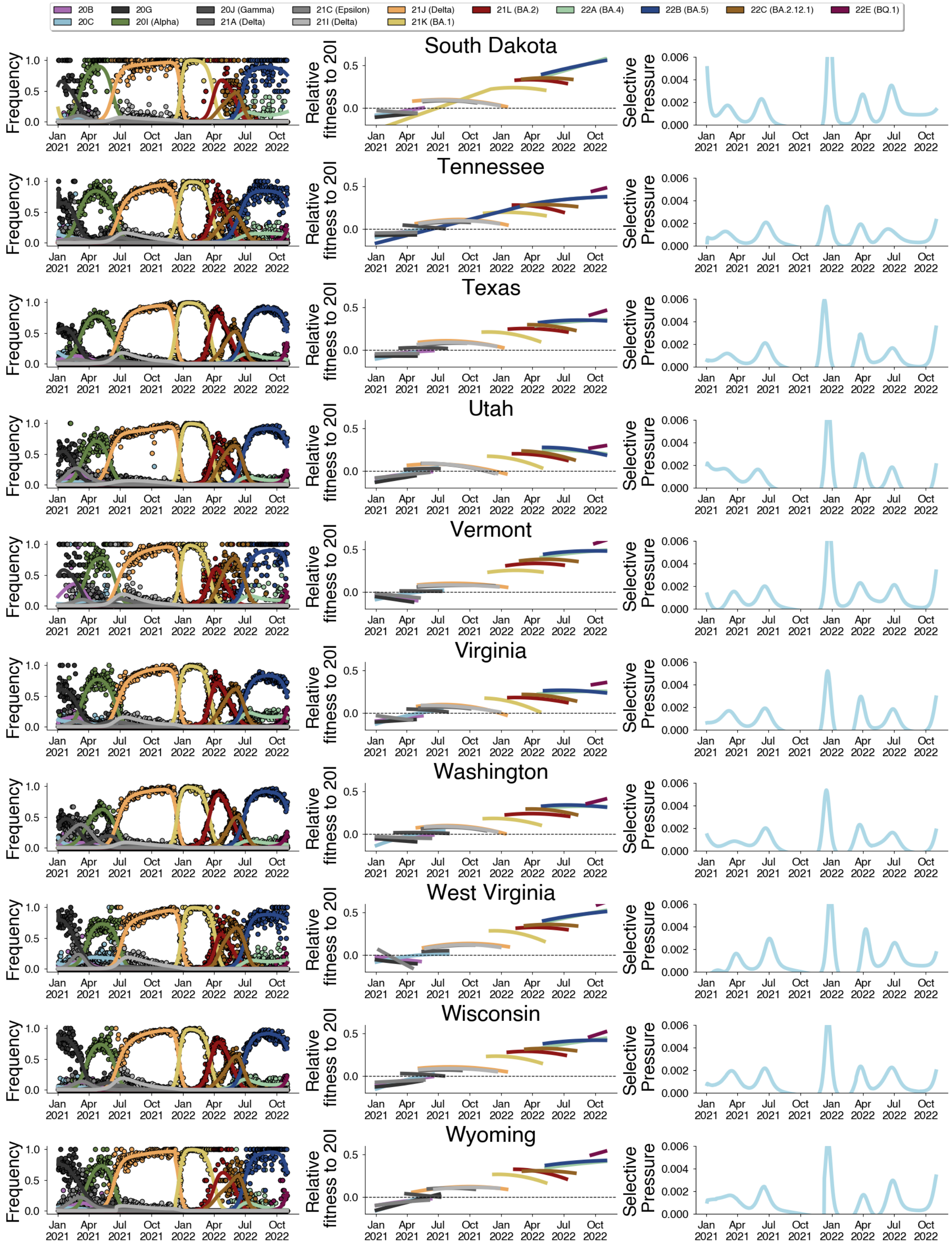

Fig. S7. Estimated variant frequencies, relative fitnesses, and selective pressure. South Dakota to Wyoming.

medRxiv preprint doi: https://doi.org/10.1101/2024.12.02.24318334; this version posted January 23, 2025. The copyright holder for this preprint (which was not certified by peer review) is the author/funder, who has granted medRxiv a license to display the preprint in perpetuity.
It is made available under a CC-BY 4.0 International license .

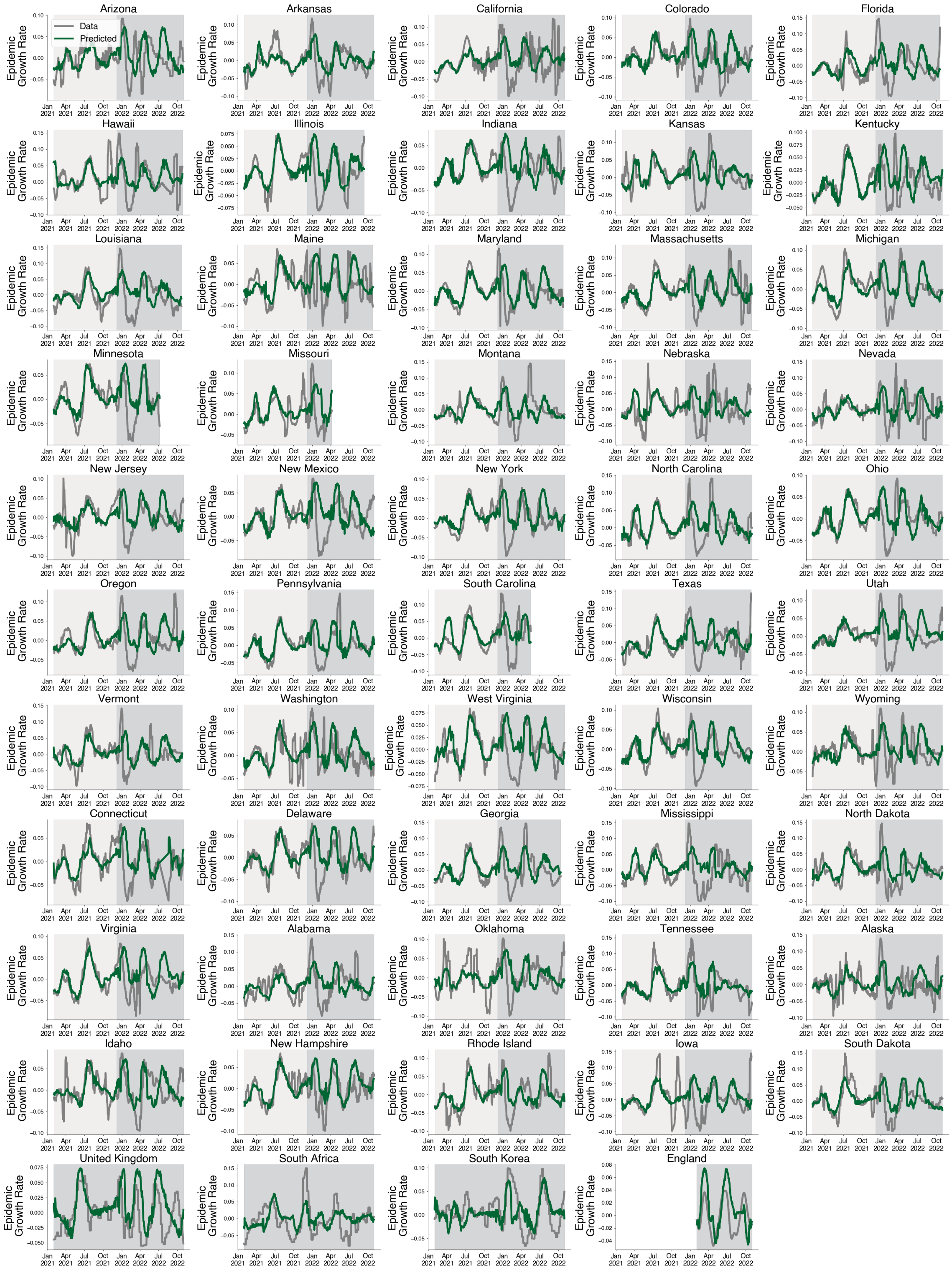

Fig. S8. Predictions for empirical growth rate using selective pressure for all locations.

medRxiv preprint doi: https://doi.org/10.1101/2024.12.02.24318334; this version posted January 23, 2025. The copyright holder for this preprint
(which was not certified by peer review) is the author/funder, who has granted medRxiv a license to display the preprint in perpetuity.
It is made available under a CC-BY 4.0 International license .

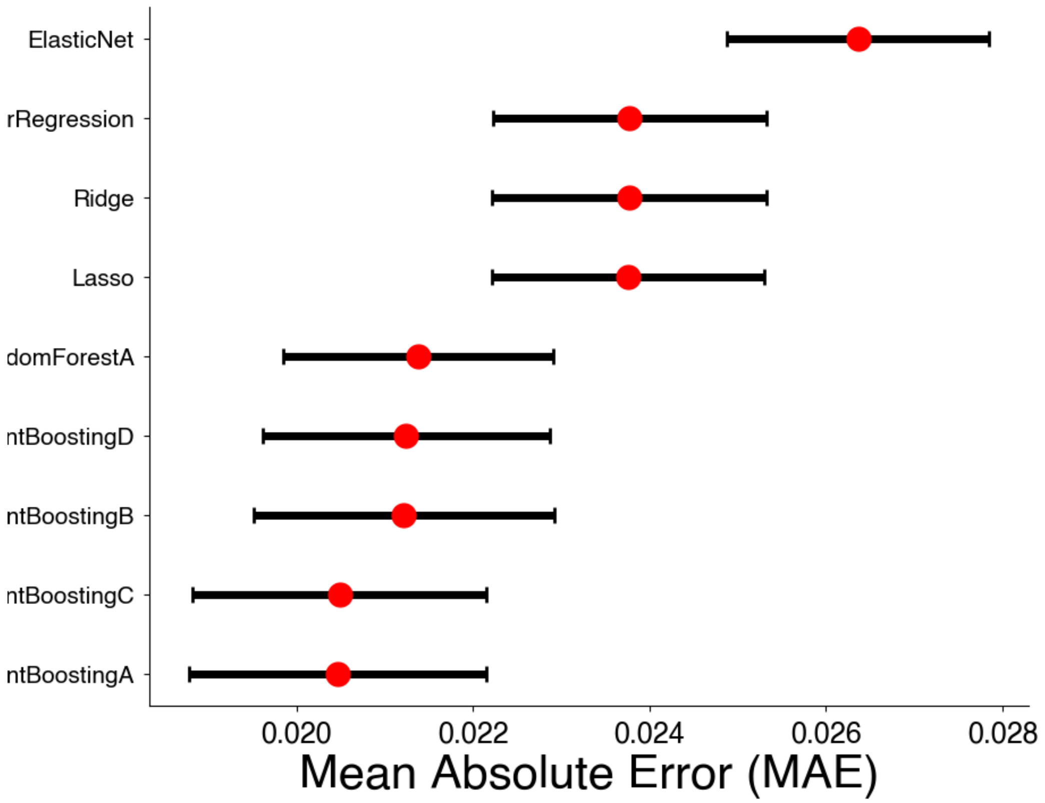

Fig. S9. Cross-validation error by model We compare the errors between models fit on 10 time series cross-validation splits.

medRxiv preprint doi: https://doi.org/10.1101/2024.12.02.24318334; this version posted January 23, 2025. The copyright holder for this preprint (which was not certified by peer review) is the author/funder, who has granted medRxiv a license to display the preprint in perpetuity.
It is made available under a CC-BY 4.0 International license .

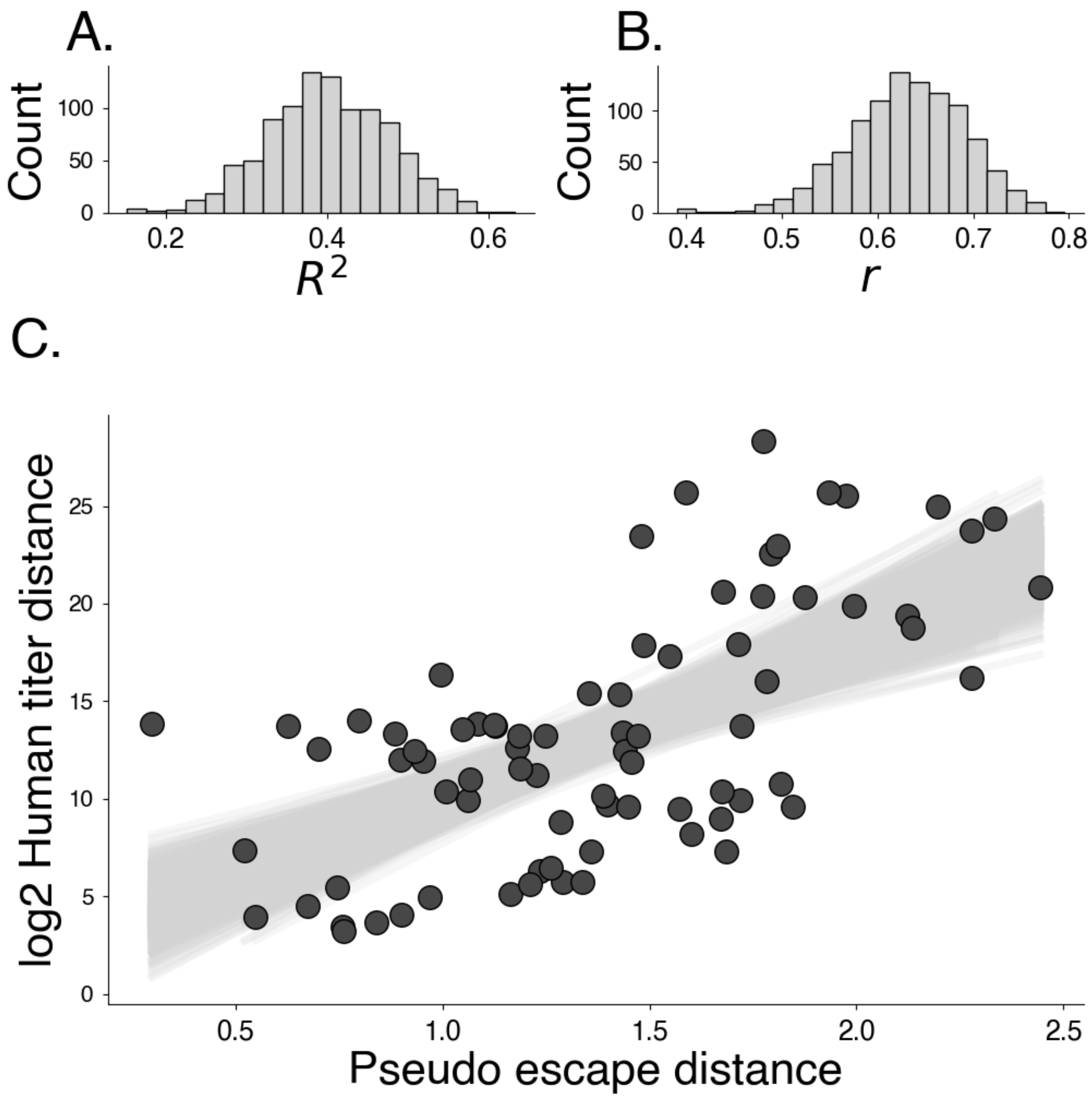

Fig. S10. Bootstrapping pseudo-immune distance and human titer distance analysis ($N_{\mathbf{replicate}} = 1,000$).

medRxiv preprint doi: https://doi.org/10.1101/2024.12.02.24318334; this version posted January 23, 2025. The copyright holder for this preprint
(which was not certified by peer review) is the author/funder, who has granted medRxiv a license to display the preprint in perpetuity.
It is made available under a CC-BY 4.0 International license .

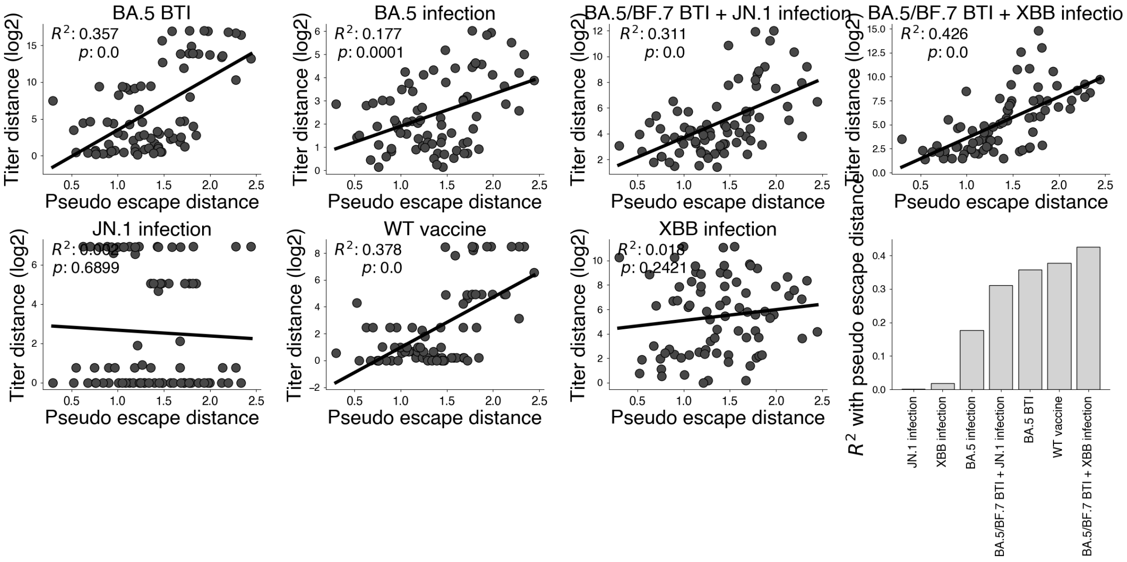

Fig. S11. Comparing pseudo-escape distance and titer distance between exposure groups.

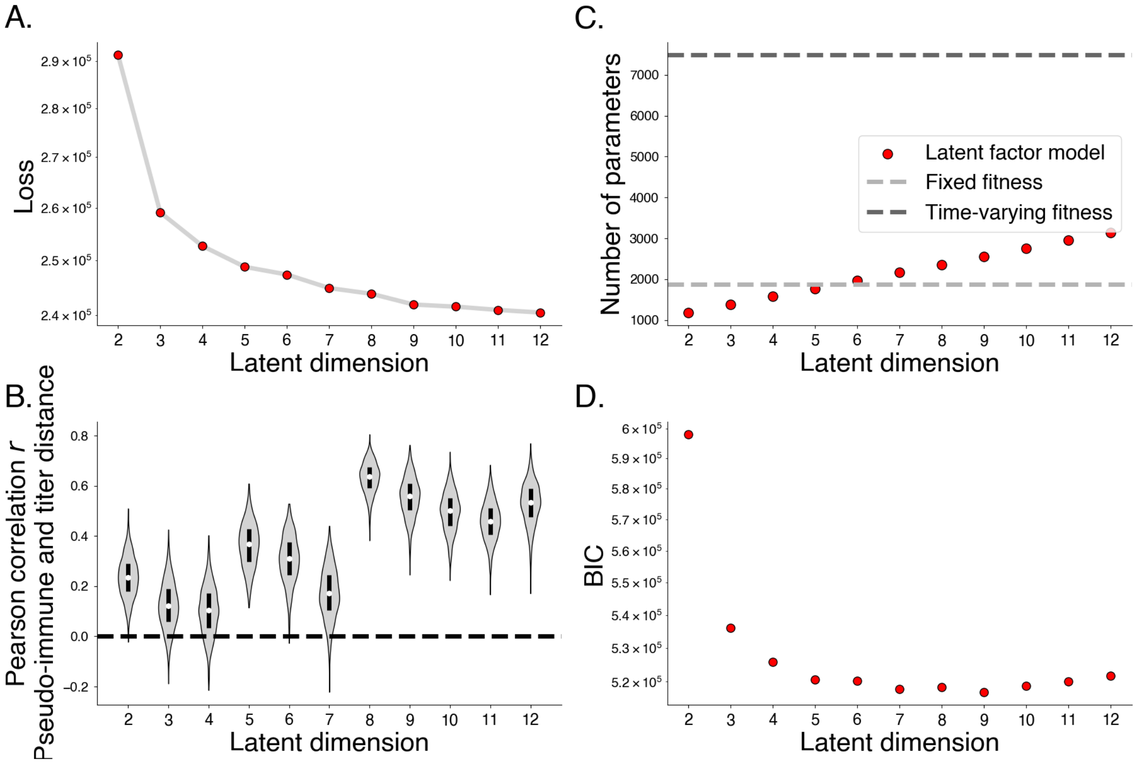

Fig. S12. Comparing latent factor model by number of latent immune dimensions. A. Maximum a posteriori loss by number of latent immune dimensions. B. Spearman correlation between pseudo-immune and titer distance by number of latent immune dimensions. C. Number of parameters by number of latent immune dimensions. D. Bayesian Information Criterion (BIC) by number of latent immune dimensions.

medRxiv preprint doi: https://doi.org/10.1101/2024.12.02.24318334; this version posted January 23, 2025. The copyright holder for this preprint (which was not certified by peer review) is the author/funder, who has granted medRxiv a license to display the preprint in perpetuity.
It is made available under a CC-BY 4.0 International license .

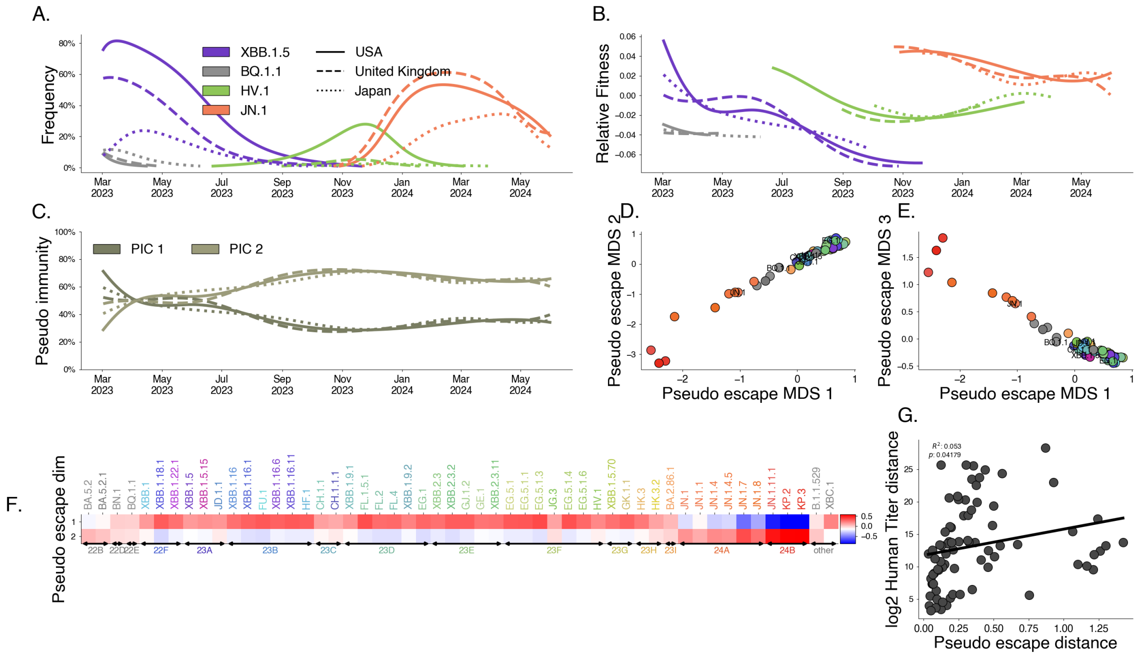

Fig. S13. Latent factor model with $D = 2$ pseudo immune dimensions.

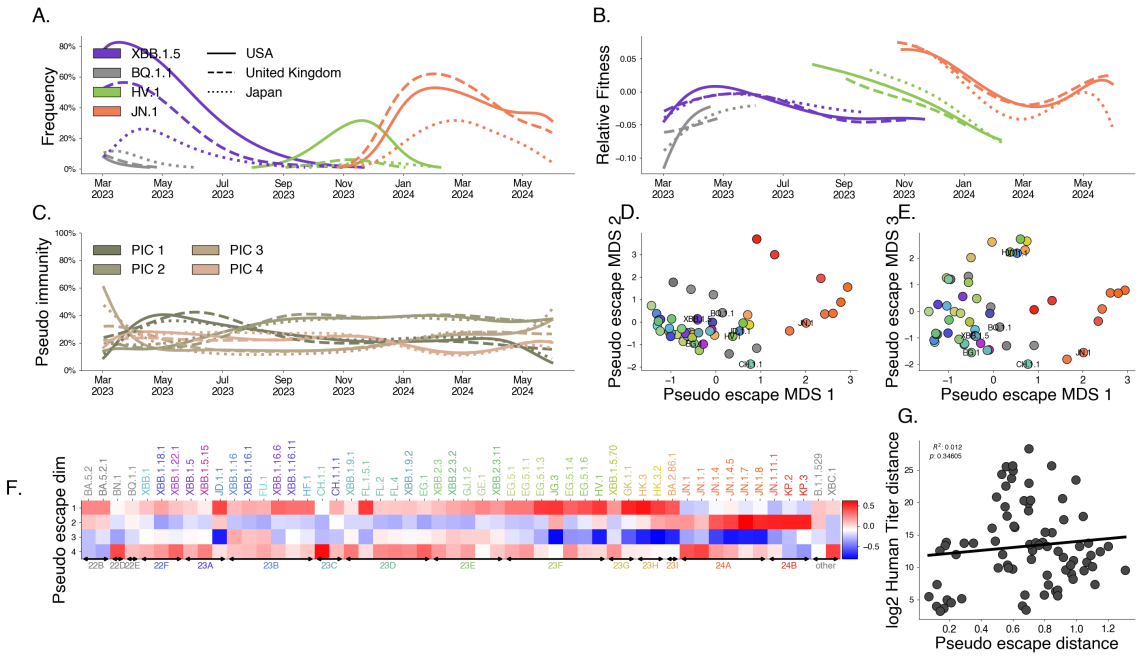

Fig. S14. Latent factor model with $D = 4$ pseudo immune dimensions.

medRxiv preprint doi: https://doi.org/10.1101/2024.12.02.24318334; this version posted January 23, 2025. The copyright holder for this preprint (which was not certified by peer review) is the author/funder, who has granted medRxiv a license to display the preprint in perpetuity.
It is made available under a CC-BY 4.0 International license .

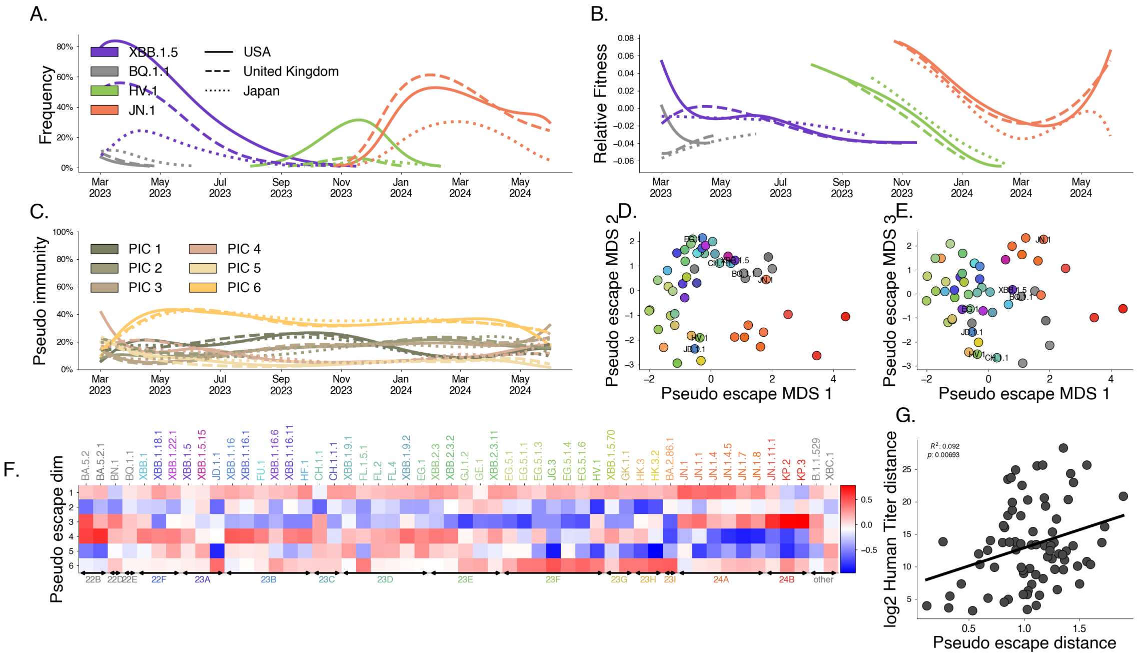

Fig. S15. Latent factor model with $D = 6$ pseudo immune dimensions.

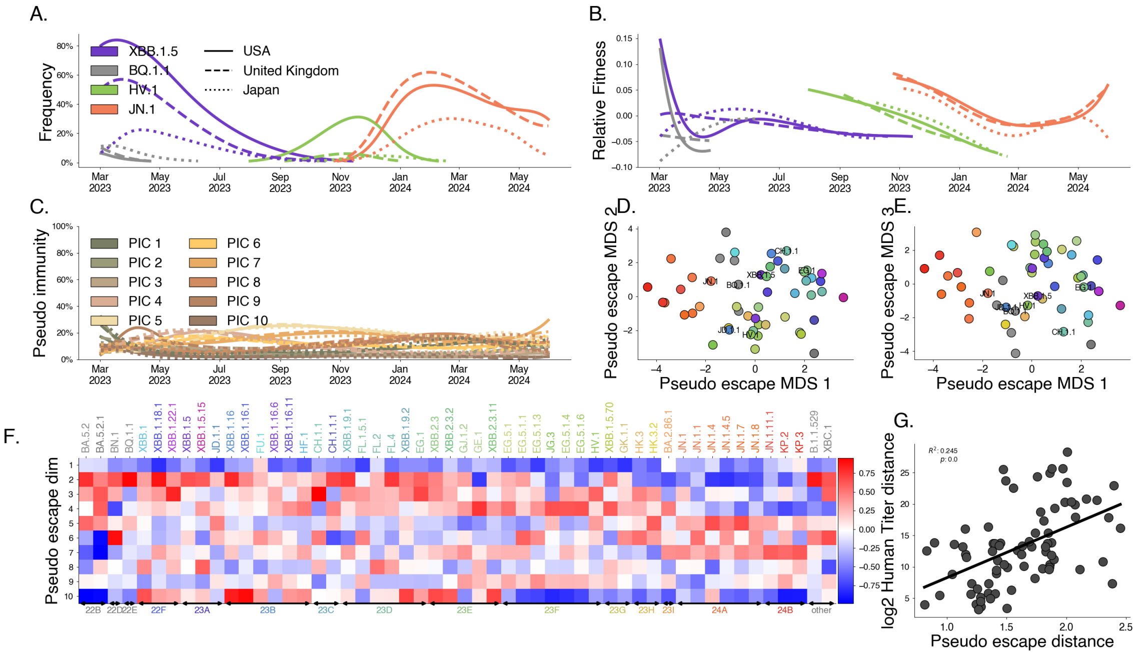

Fig. S16. Latent factor model with $D = 10$ pseudo immune dimensions.
