# Supplementary material for: Frequency dynamics predict viral fitness, antigenic relationships and epidemic growth
Source: medRxiv. 2024 Dec 3:2024.12.02.24318334. Preprint. [Version 1] doi: 10.1101/2024.12.02.24318334 (PMC11643185; doi:10.1101/2024.12.02.24318334)
Supplement: Supplementary file 1 [file NIHPP2024.12.02.24318334v1-supplement-1.pdf]

medRxiv preprint doi: https://doi.org/10.1101/2024.12.02.24318334; this version posted December 3, 2024. The copyright holder for this preprint (which was not certified by peer review) is the author/funder, who has granted medRxiv a license to display the preprint in perpetuity.
It is made available under a CC-BY 4.0 International license .

Supplementary Information for

Frequency dynamics predict viral fitness, antigenic relationships and epidemic growth

Materials and Methods

Generating sequence counts We prepared sequence count data sets using the Nextstrain-curated SARS-CoV-2 sequence metadata [31] which is created using the GISAID EpiCoV database [32]. These sequences were tallied according to either their annotated Nextstrain clade or Pango lineage [12] depending on the data set to produce sequence count for each variant, for each day over the period of interest, and in each country analyzed.

Likelihood of sequence counts given frequencies The models discussed in this paper use observed counts of variant sequences to inform the underlying variant frequency in the population. This is accomplished using a multinomial likelihood, so that given count of sequences $S_v(t)$ of variant v at time t and total sequences $N(t)$ collected at time t, we have that

$$S_v(t) \sim \text{Multinomial}(N(t), f_v(t)),$$

where $f_v(t)$ is the frequency of variant v at time t. This is a simple model of sequence counts to frequencies and does not account for over-dispersion of sequence counts relative to a multinomial. However, all models can be extended to estimate and account for over-dispersion by replacing the above likelihood with a Dirichlet-Multinomial likelihood.

Approximate Gaussian processes for relative fitness estimation To generate smooth non-parametric estimates of variant growth rates, we develop a Gaussian process based model for relative fitnesses. That is, we model the relative fitness for each variant over time $\lambda_v(t)$ as a multivariate normal distribution:

$$\begin{aligned} \boldsymbol{\lambda}_v &\sim \text{Normal}(\boldsymbol{\mu}, \boldsymbol{\Sigma}) \\ \boldsymbol{\Sigma}_{s,t} &= K_\theta(s,t), \end{aligned}$$

where K_θ is a potentially parameterized kernel function. This induces a structure on the covariance of the relative fitness values over time points s and t.

For computational efficiency, we implement a Hilbert Space Gaussian Process (HSGP) approximation instead of fitting V independent Gaussian processes. This approximation allow us to share basis functions between variants [26]. Under this approximation, the relative fitnesses are computed as

$$\lambda_v(t) \approx \sum_{j=1}^{m} S_\theta(\sqrt{\mu_j})^{1/2} \cdot \phi_j(t) \cdot \beta_j, \tag{7}$$

where S_θ is the spectral density of the kernel K_θ, μ_j and ϕ_j are the eigenvalues and eigenfunctions of the Laplacian, and $\beta_j \sim \text{Normal}(0,1)$ [26]. Since the eigenvalues and

medRxiv preprint doi: https://doi.org/10.1101/2024.12.02.24318334; this version posted December 3, 2024. The copyright holder for this preprint (which was not certified by peer review) is the author/funder, who has granted medRxiv a license to display the preprint in perpetuity.
It is made available under a CC-BY 4.0 International license .

eigenfunctions are shared across variants, this allows us to re-use values across variants, simplifying the computation to a matrix multiplication as

$$\boldsymbol{\lambda}_t = \boldsymbol{\Phi}_t \sqrt{\boldsymbol{S}_\theta} \boldsymbol{\beta}.$$

For the analyses in this paper, we use this approximate Gaussian process with a Matérn 5/2 kernel and shared hyperparameters across variants. We demonstrate this model for simulated data from Fig. 1 and show resulting relative fitnesses through time in Fig. S1.

Correlations are insufficient for mechanism identification To assess how vaccination uptake affects the growth advantage of a variant with increased transmissibility, we simulate the spread of a more transmissible variant across populations with different initial past exposure and vaccination levels. This enables us to isolate the effects of transmissibility within different immunity landscapes, examining how relative fitness and growth advantage shift based on population vaccination coverage alone in the absence of immune escape. We begin with the 2-variant SIR model described in Supplementary Text S1. We simulate this model for 100 days with generation time $\tau = 1/\gamma = 3.0$ days, $R_{0,W} = 1.4$, $I_W(0) = 100$ individuals, $I_v(0) = 1$ individual, a 50% transmissibility increase $\rho = 0.5$, and no immune escape $\eta = 0.0$. We divide the period into early and late epidemic with the breakpoint being $t = 50$. In Fig. 3D-E, we estimate the log growth advantage for the variant in the early and full periods using a logit-linear model:

$$\log\left(\frac{f_v(t)}{1 - f_v(t)}\right) = \beta t/\tau + \alpha,$$

where we take the model slope β to be our log growth advantage.

We repeat these simulations for a range of vaccination levels starting from 0% and ending at 65%.

Predicting epidemic growth rate from selective pressure The derivation of the selective pressure metric shows that the selective pressure can be a useful tool in predicting the epidemic growth rate. To develop a predictive model of epidemic growth rate using selective pressure, we begin by generating estimates of selective pressure and epidemic growth rate from a period with high sequencing and case surveillance.

We take sequence count and case count data from all states in the United States between January 2021 and November 2022. State-level daily case counts were obtained from USAFacts downloaded on August 7, 2024 at https://usafacts.org/visualizations/coronavirus-covid-19-spread-map/.

Using the sequence counts, we compute selective pressure estimates from relative fitness and frequencies estimated with our approximate Gaussian process relative fitness model. From the case data, we derive the empirical growth rate using a 14-day moving average on case counts $\hat{C}_t$ and computing the empirical growth rate as $\hat{r}_t = \log(\hat{C}_t) - \log(\hat{C}_{t-1})$. We then use the past 28 days of selective pressure to predict the empirical growth rate.

medRxiv preprint doi: https://doi.org/10.1101/2024.12.02.24318334; this version posted December 3, 2024. The copyright holder for this preprint (which was not certified by peer review) is the author/funder, who has granted medRxiv a license to display the preprint in perpetuity.
It is made available under a CC-BY 4.0 International license .

We use a gradient boosting regressor model which is fit using a mean absolute error loss function. This model was selected as it achieved the minimal error via time series cross-validation averaged across 10 splits among candidate models (Fig. S9). The candidate models include linear regression, ridge regression, Lasso regression, random forests, and gradient-boosted trees as implemented in scikit-learn [33]. We additionally tune the hyperparameters of this model using grid search cross-validation.

We validate our model by comparing our predicted epidemic growth rates to held-out case data for US states, and additionally to estimates of the epidemic growth rates in England derived from data from the Office for National Statistics (ONS) Coronavirus Infection Survey [30]. Estimates of prevalence from the ONS Infection Survey were obtained for January 2022 to September 2022 from www.ons.gov.uk/peoplepopulationandcommunity/healthandsocialcare/conditionsanddiseases/datasets/coronaviruscovid19infectionsurveydata. Epidemic growth rates are computed on this data in the same way as the state-level analysis.

Latent immune factor model We show that relative fitness dynamics can be explained by low-dimensional immunity when transmission dynamics are described with compartmental models (Supplementary Text S1). This motivates a model to learn this low-dimensional structure that is inspired by latent-factor models. We start by assuming that the relative fitness of variant v at time t and in geographic location g can be described by D latent factors so that

$$\lambda_v^g(t) = \sum_{d=1}^{D} \eta_{v,d}\, \phi_d^g(t). \tag{8}$$

As the structure here resembles Equation 14, we call $\eta_{v,d}$ "pseudo-escape" of variant v from group d and ϕ_d^g "pseudo-immunity" group d in geographic location g. To make this more consistent with our intuition here, we model ϕ_d^g to be in $[0, 1]$ and model it as smoothly varying in time. We model $\text{logit}(\phi_d^g)$ using 4th order splines with 6 knots placed uniformly over the time period modeled. Though we choose to model these latent factors with splines, other models would work here. For example, one alternative would be the approximate Gaussian processes described above. Additionally, in order to ensure identifiability of the parameter estimates, we fix some base variant v^* which fitness is defined relative to, so that $\eta_{v^*,d} = 0$ for all $1 \leq d \leq D$. For the same reason, we fix the order of components, so that the components are numbered in decreasing order by their share in the arbitrarily defined base geography.

We apply this model to SARS-CoV-2 sequence counts in the period between March 2023 to March 2024 for 14 countries. To access the necessary number of immune dimensions, we vary the number of immune dimensions between $D = 2$ to $D = 12$. Looking at the loss for the latent factor model for increasing D, we choose $D = 10$ for our primary analysis by noting the point at which the loss function seems to stagnate with increasing D i.e. the "elbow" method (Fig. S12).

We compare the distances between variant pairs in our estimated pseudo-escape space to distances in log2 titer. Using human titer data from Jian et al [7], we compute neutraliza-

medRxiv preprint doi: https://doi.org/10.1101/2024.12.02.24318334; this version posted December 3, 2024. The copyright holder for this preprint (which was not certified by peer review) is the author/funder, who has granted medRxiv a license to display the preprint in perpetuity.
It is made available under a CC-BY 4.0 International license .

tion titer distances as the average of differences in log2 neutralization titers between pairs of variants for a cohort of individuals. This analysis is repeated among 1,000 bootstrapped samples to create a distribution of R^2 values (Fig. S10). Additionally, we subset this by exposure history and repeat this analysis to find which exposure groups best explain distances in pseudo-escape space (Fig. S11).

Data and code accessibility

Source code used to generate figures, model implementations, and sequence count data are available at github.com/blab/relative-fitness-mechanisms.

medRxiv preprint doi: https://doi.org/10.1101/2024.12.02.24318334; this version posted December 3, 2024. The copyright holder for this preprint (which was not certified by peer review) is the author/funder, who has granted medRxiv a license to display the preprint in perpetuity.
It is made available under a CC-BY 4.0 International license .

Supplementary Text

S1 Exponentially growing populations to frequency dynamics

We consider a viral population consisting of V exponentially-growing variant viruses each with prevalence I_v. Defining the time-varying growth rate for the prevalence of variant v as $r_v(t)$, we can represent the prevalence using the following ordinary differential equation:

$$\frac{dI_v}{dt} = r_v(t)I_v(t), \quad v = 1, 2, \ldots, V. \tag{9}$$

The above differential equation has a known solution in terms of the integral of the time-varying growth rate and initial prevalence as follows:

$$I_v(t) = I_v(0)\exp\left(\int_0^t r_v(s)ds\right),$$

where $I_v(0)$ is the initial prevalence of variant v.

Now turning to the frequency dynamics of the population, we write the frequency of variant v in the population as $f_v(t) = I_v(t)/\sum_{u=1}^{V} I_u(t)$. This allows us to derive an ODE for variant frequency in terms of the variant growth rates using the quotient rule for differentiation:

$$\begin{aligned}\frac{df_v}{dt} &= f_v\left(\sum_{u=1}^{V}[r_v(t) - r_u(t)]f_u\right)\\ &= f_v\left(r_v(t) - \sum_{u=1}^{V} r_u(t)f_u\right).\end{aligned}$$

This system of differential equations resembles a logistic growth equation and can be shown to have the following solution in terms of the initial frequencies $f_v(0)$ and the variant growth rates:

$$f_v(t) = \frac{f_v(0)\exp(\int_0^t r_v(s)ds)}{\sum_{u=1}^{V} f_u(0)\exp(\int_0^t r_u(s)ds)}. \tag{10}$$

The above representation of the variant frequency will serve as a centerpiece for many of the arguments to follow. We see that by tracking the rate at which variant viruses are spreading, we can construct the corresponding frequency dynamics without knowing the absolute prevalence of any variant.

Relative frequency and relative fitness Using the above equation for the variant frequencies, we can write the relative frequency of variant v over u as $x_{v,u}(t) = f_v(t)/f_u(t)$

medRxiv preprint doi: https://doi.org/10.1101/2024.12.02.24318334; this version posted December 3, 2024. The copyright holder for this preprint (which was not certified by peer review) is the author/funder, who has granted medRxiv a license to display the preprint in perpetuity.
It is made available under a CC-BY 4.0 International license .

to see:

$$x_{v,u}(t) = \frac{f_v(t)}{f_u(t)} = \frac{f_v(0)}{f_u(0)} \exp\left(\int_0^t [r_v(s) - r_u(s)]ds\right)$$
$$= x_{v,u}(0) \exp\left(\int_0^t \lambda_{v,u}(s)ds\right).$$

Notice this relative frequency change depends on the initial relative frequencies and the *relative fitness* $\lambda_{v,u}(t) = r_v(t) - r_u(t)$ of v over u. This relative fitness has the same units as the exponential growth rate (e.g. per day). Using the definition of relative fitness, we can notice that

$$\lambda_{v,u}(t) = r_v(t) - r_u(t) = \frac{d}{dt}\left[\log\left(x_{v,u}(t)\right)\right] = -\lambda_{u,v}(t) \tag{11}$$

We can see that there is a symmetry in the relative fitnesses and that the associated frequency dynamics depend on the differences between relative fitnesses. This suggests that absolute fitness (in terms of the growth of infections) may not be inferable from frequencies alone. This definition of relative fitness becomes essential in describing various existing modeling approaches for frequency dynamic data and motivates possible extensions since we can represent these models as having the form:

$$f_v(t) = \frac{f_v(0)\exp(\int_0^t \lambda_{v,v^*}(s)ds)}{\sum_{u=1}^{V} f_u(0)\exp(\int_0^t \lambda_{u,v^*}(s)ds)}, \tag{12}$$

where the growth rate of v is expressed as relative to an arbitrary pivot variant v^*.

Cumulative relative-fitness and frequency change Above we saw that within our framework frequency change over time intervals depends only on the cumulative relative fitness over time intervals $\Lambda_{v,u}(0,t) = \int_0^t \lambda_{v,u}(s)ds$. We can then characterize approaches for modeling frequency change in terms of how they represent, estimate, and forecast these relative fitnesses. This framework includes various existing methods for analyzing frequency data such as the seasonal influenza forecasting models of Lässig and Łuksza [34] and Huddleston et al [35], multinomial logistic regression for frequency estimation [13] and the SARS-CoV-2 mutational fitness model of Obermeyer et al [36].

Though this framework can be used to describe existing statistical methods for frequency modeling, it is also applicable to traditional compartmental models of epidemics. In fact, applying these ideas to compartmental models enables to see how mechanistic assumptions on the transmission process determine relative fitness of variant viruses.

Two-strain SIR For simplicity, we will begin by analyzing a two-strain SIR model in which a variant virus v can differ from wildtype virus wt by increased intrinsic transmissibility (via η_T) and immune escape against wild-type immunity (via η_E). This system of 5 ordinary differential equations follows

medRxiv preprint doi: https://doi.org/10.1101/2024.12.02.24318334; this version posted December 3, 2024. The copyright holder for this preprint (which was not certified by peer review) is the author/funder, who has granted medRxiv a license to display the preprint in perpetuity.
It is made available under a CC-BY 4.0 International license .

$$\begin{aligned}
\frac{dS}{dt} &= -\beta S I_W - \beta\rho S I_v \\
\frac{dI_W}{dt} &= \beta S I_W - \gamma I_W \\
\frac{dI_v}{dt} &= \beta(1+\rho) S I_v + \beta(1+\rho)\eta\phi_W I_v - \gamma I_v \\
\frac{d\phi_W}{dt} &= \gamma I_W - \beta(1+\rho)\eta\phi_W I_v \\
\frac{d\phi_v}{dt} &= \gamma I_v.
\end{aligned}$$

where I_W denotes wild-type prevalence, I_v denotes variant prevalence and ϕ_W denotes immunity derived from wild-type infection. In this model, the variant virus can infect both susceptible individuals S and individuals with immunity to wild-type virus ϕ_W. Increased intrinsic transmissibility increases the baseline transmission rate from β in wild-type to $\beta\rho$ in the variant virus and immune escape increases the transmission rate against those with wildtype immunity, so that the at-risk population is $\eta\phi_W$.

Writing that $r_W(t) = \beta S - \gamma$ and $r_v(t) = \rho\beta S + \beta(1+\rho)\eta\phi_W - \gamma$, we can then write the relative fitnesses as:

$$\lambda_{v,W}(t) = \rho\beta S(t) + (1+\rho)\eta\beta\phi_W(t). \tag{13}$$

From this representation of relative fitness, we can see that given fixed increases to overall transmission ($\rho > 0$) or immune escape ($\eta > 0$), the observed fitness boost at the level of variant relative fitness still depends on the proportion of the population at risk for infection.

n-strain SIR This model can also be extended to an n-strain SIR model where each variant strain v_i with $2 \leq i \leq n$ is described by its own advantage parameters $\theta_i = (\rho^{(i)}, \eta^{(i)})$ relative to the wildtype ($\theta_W = \theta_1 = (0,0)$).

$$\begin{aligned}
\frac{dS}{dt} &= -\beta S I_W - \beta(1+\rho) S I_{v_i} \\
\frac{dI_W}{dt} &= \beta S I_W - \gamma I_W \\
\frac{dI_{v_i}}{dt} &= \beta(1+\rho_i) S I_{v_i} + \beta(1+\rho_i)\eta_i\phi_W I_{v_i} - \gamma I_{v_i} \\
\frac{d\phi_W}{dt} &= \gamma I_W - \beta(1+\rho_i)\eta_i\phi_W I_{v_i} \\
\frac{d\phi_{v_i}}{dt} &= \gamma I_{v_i}, \quad i \in \{2, \ldots, n\}.
\end{aligned}$$

In this formulation, the variant viruses compete only for susceptible population and those with previous wild-type infection. This formulation can be generalized to allow for competition between all variants for any exposure history and will be discussed in the following sections. In Fig. 1, we implement and simulate a 3-strain model with wildtype as above, an escape variant E with $\theta_2 = (0, \eta)$, and a transmissibility increase variant T with $\theta_3 = (\rho, 0)$.

medRxiv preprint doi: https://doi.org/10.1101/2024.12.02.24318334; this version posted December 3, 2024. The copyright holder for this preprint (which was not certified by peer review) is the author/funder, who has granted medRxiv a license to display the preprint in perpetuity.
It is made available under a CC-BY 4.0 International license .

Models of immune escape against heterogeneous backgrounds We'll now consider a model where all hosts are assumed to fall into one of B immune backgrounds ϕ_b for $b = 1, \dots, B$. We assume that infection by each variant v then leaves recovered hosts in the corresponding immune background of the most recent infection b_v. Variant transmission then occurs via immune escape against a background leading to a matrix of escape rates $\boldsymbol{\eta} = \eta_{v,b}$ for variants v and background b.

We can then write the system of ordinary differential equations as

$$\frac{dI_v}{dt} = \beta \sum_{1 \leq b \leq B} \eta_{v,b}\phi_b I_v - \gamma I_v, \quad v = 1, \dots, V$$

$$\frac{d\phi_b}{dt} = -\beta \sum_{1 \leq v \leq V} \eta_{v,b}\phi_b I_v + \sum_{v:\ b_v = b} \gamma I_v.$$

With this model, susceptible and recovered compartments in the standard SIR model can be thought of as immune backgrounds. This allows us to represent the standard SIR model as $S = \phi_S$, $I = I_W$, $R = \phi_W$ and $\eta_{W,S} = 1, \eta_{W,W} = 0$ and $b_W = W$. We can also think of the two-strain SIR with $\rho = 1$ as a special case of this model where we set $S = \phi_S, \eta_{W,S} = 1, \eta_{W,W} = 0, \eta_{v,S} = 1, \eta_{v,W} = \eta$ and keep all other parameters the same.

With this formulation of immune escape, we can then write the relative fitnesses in terms of the escape rates $\eta_{v,b}$ and the immune background proportions ϕ_b as

$$\lambda_{v,u}(t) = \beta \sum_{1 \leq b \leq B} (\eta_{v,b} - \eta_{u,b})\phi_b(t). \tag{14}$$

Under this model of immune escape, we can see relative fitness among variants can be decomposed into differences in immune escape among immune backgrounds within a population. Due to the dependence here on the proportion of each immune background in determining fitness, this suggests that the overall distribution of susceptibility to strains is potentially an important consideration when translating individual-level measures of immune escape to population-level estimates of variant fitness. Understanding the size and complexity of this immune space may therefore be useful for parameterization and forecasting of variant frequencies. However, the extent to which modeling this complexity affects estimates of relative fitness also depends on how quickly the distribution of immune backgrounds change i.e. $\frac{d\phi_b}{dt}$.

Though the derivation above uses a simplified model of using most recent infection to sort individuals into an immune group, we show that a more complicated model that accounts for the entire exposure history of the host also gives a similar decomposition to relative fitness in Supplementary Text S3.

S2 Revisiting existing models for frequency growth

Using the theory developed for exponentially-growing variant populations, we now re-visit existing methods for modeling viral frequency dynamics.

medRxiv preprint doi: https://doi.org/10.1101/2024.12.02.24318334; this version posted December 3, 2024. The copyright holder for this preprint (which was not certified by peer review) is the author/funder, who has granted medRxiv a license to display the preprint in perpetuity.
It is made available under a CC-BY 4.0 International license .

Multinomial Logistic Regression We begin with multinomial logistic regression (MLR) with fixed relative fitness. This model can be written as

$$f_v(t) = \frac{f_v(0)\exp(\lambda_v t)}{\sum_u f_u(0)\exp(\lambda_u t)},$$

where $f_v(t)$ is the frequency of variant v at time t and λ_v is the relative fitness of variant v. This provides estimates of the relative fitness compared to some reference strain u^* for which $\lambda_{u^*} = 0$. In this model, initial frequencies $f_v(0)$ and relative fitness λ_v are estimated from frequency dynamics. Converting this estimate to an estimate of transmission advantage (relative effective reproduction number) requires assuming a delta distribution of the generation time [37].

Comparing this to equation 13, we can see this model of fixed relative fitness results from assuming that the at-risk populations are constant over-time. This assumption is useful since it requires no outside knowledge of the at-risk population and relative infection rates, though this may be less useful for longer forecasts or when there is large turnover in at-risk populations due to infection.

Fitness models of seasonal influenza Motivated by the observed antigenic evolution of seasonal influenza, Lässig and Łuksza [34] and Huddleston et al [35] approximate the cumulative relative fitness between influenza seasons on the level of individual strains as

$$\Lambda_{v,u}(t + \Delta t, t) = (\beta_1 x_{v,1} + \cdots + \beta_p x_{v,p})\Delta t = (\boldsymbol{\beta} \cdot \boldsymbol{x}_v)\Delta t,$$

where the relative fitness is determined by strain-specific predictors $\boldsymbol{x}_v$ and the regression parameter $\boldsymbol{\beta}_v$ are estimated.

This formulation fits neatly into the framework we've developed as the cumulative fitness here can be written as the integral of a relative fitness $\lambda_{v,u} = \boldsymbol{\beta} \cdot \boldsymbol{x}_v$ over the time period of interest:

$$\Lambda_{v,u}(t + \Delta t, t) = \int_t^{t+\Delta t} \lambda_{v,u}(s)ds \ = \int_t^{t+\Delta t} (\boldsymbol{\beta} \cdot \boldsymbol{x}_v)ds.$$

Therefore, these models can be thought as regression-based predictors of relative fitness where frequency and external covariates contribute to estimated relative fitness.

S3 Relative fitness for full immune history models

We show that the simple background model is consistent with an expanded immune history model. Beginning with the model from Lazebnik and Bunimovich-Mendrazitsky 2022 [38],

medRxiv preprint doi: https://doi.org/10.1101/2024.12.02.24318334; this version posted December 3, 2024. The copyright holder for this preprint (which was not certified by peer review) is the author/funder, who has granted medRxiv a license to display the preprint in perpetuity.
It is made available under a CC-BY 4.0 International license .

we consider the differential equation for the individuals with strain infection history J and current infecting strain i $R_J I_i$

$$\frac{dR_J I_i}{dt} = -\gamma_{J,i} R_J I_i + \beta_{J,i} R_J \sum_{K \in P(M), i \notin K} R_K I_i.$$

Here, infection can occur from any individual infected with strain i assuming their past immune history does not include i and the infected are any recovered individual with immune history J R_J. To compute the strain growth rate, we can sum over all possible immune histories for individuals infected with strain i, so that

$$\begin{aligned}
\frac{dI_i}{dt} &= \sum_{J \in P(M), i \notin J} \frac{dR_J I_i}{dt} \\
&= -\gamma_i I_i + \sum_{J \in P(M), i \notin J} \beta_{i,J} R_J \sum_{K \in P(M), i \notin K} R_K I_i \\
&= -\gamma_i I_i + \sum_{J \in P(M), i \notin J} \beta_{i,J} R_J I_i \\
&= \left(-\gamma_i + \sum_{J \in P(M), i \notin J} \beta_{i,J} R_J \right) I_i \\
&= \left(-\gamma + \beta \sum_{J \in P(M), i \notin J} \eta_{i,J} R_J \right) I_i
\end{aligned}$$

Assuming that the transmission rate can be decomposed as a base transmission rate β and a strain i and immune history J specific escape rate $\eta_{i,J}$ and that the recovery rate is constant, we notice this is identical to our previous immune background model. Therefore, our relative fitnesses can be written as

$$\lambda_{i,j} = \beta \sum_{B \in P(M)} (\eta_{i,B} - \eta_{j,B}) R_B,$$

where for simplicity we define $\eta_{v,B} = 0$ if $v \in B$.

S4 Selective pressure and contribution to epidemic growth rates

In this section, we derive our selective pressure metric $\psi(t)$ and show how it contributes to the overall epidemic growth rate in the population.

Beginning again from our assumption of inhomogeneous exponential growth, we can write a differential equation for the total prevalence $I(t) = \sum_v I_v(t)$ as follows:

medRxiv preprint doi: https://doi.org/10.1101/2024.12.02.24318334; this version posted December 3, 2024. The copyright holder for this preprint (which was not certified by peer review) is the author/funder, who has granted medRxiv a license to display the preprint in perpetuity.
It is made available under a CC-BY 4.0 International license .

$$
\begin{aligned}
\frac{dI}{dt} &= \sum_v \frac{dI_v}{dt} = \sum_v r_v(t) I_v(t) \\
&= \left(\sum_v r_v(t) f_v(t) \right) I(t),
\end{aligned}
$$

where we've used that $I_v(t) = f_v(t)I(t)$. This allows us to see that $\overline{r}(t) = \sum_v r_v(t)f_v(t)$ is the average growth rate of the prevalence. Re-writing the average in terms of some base exponential growth rate and the relative fitnesses so that $r_v(t) = \lambda_v(t) + r_W(t)$, we get that $\overline{r}(t) = \sum_v \lambda_v(t)f_v(t) + r_W(t)$. We can simplify this by writing $\overline{r}(t) = \overline{\lambda}(t) + r_W(t)$ where $\overline{\lambda}(t) = \sum_v \lambda_v(t)f_v(t)$ is the mean fitness of the population. We can now look at the rate of change in the average growth rate by taking its derivative.

$$
\begin{aligned}
\frac{d\overline{r}}{dt} &= \frac{dr_W}{dt} + \frac{d\overline{\lambda}}{dt} \\
&= \frac{dr_W}{dt} + \sum_v \left[\frac{d\lambda_v}{dt} f_v(t) + \lambda_v(t) \frac{df_v}{dt} \right] \\
&= \frac{dr_W}{dt} + \sum_v \left[\frac{d\lambda_v}{dt} f_v + \lambda_v f_v (\lambda_v - \overline{\lambda}) \right] \\
&= \frac{dr_W}{dt} + \sum_v \frac{d\lambda_v}{dt} f_v(t) + \sum_v \lambda_v (\lambda_v - \overline{\lambda}) f_v \\
&= \frac{dr_W}{dt} + \sum_v \frac{d\lambda_v}{dt} f_v(t) + \sum_v \lambda_v^2 f_v - \overline{\lambda} \sum_v \lambda_v f_v \\
&= \frac{dr_W}{dt} + \mathbb{E}_{f(t)} \left[\frac{d\lambda_v}{dt} \right] + \mathbb{V}_{f(t)}[\lambda_v].
\end{aligned}
$$

Here, we've written the last line in terms of expectations relative to sampling according to the frequency distribution. This shows us that the change in the average growth rate of the epidemic can be written in terms of the growth rate of the pivot category, the mean rate of change in the relative fitness, and the variance of the relative fitnesses. We will call terms which can be computed in terms of quantities derived from frequencies alone the selective pressure and denote it as

$$
\psi(t) = \mathbb{E}_{f(t)} \left[\frac{d\lambda_v}{dt} \right] + \mathbb{V}_{f(t)}[\lambda_v]. \tag{15}
$$

We can use this idea to directly write the prevalence in terms of the selective pressure and the base growth rate. First, we define a cumulative selective pressure as

$$
\Psi(t) = \int_0^t \psi(s) ds.
$$

medRxiv preprint doi: https://doi.org/10.1101/2024.12.02.24318334; this version posted December 3, 2024. The copyright holder for this preprint (which was not certified by peer review) is the author/funder, who has granted medRxiv a license to display the preprint in perpetuity.
It is made available under a CC-BY 4.0 International license .

We can then use this to reconstruct the relative incidence as:

$$\begin{aligned}\frac{I(t)}{I(0)} &= \exp\left(\int_0^t \overline{r}(s)ds\right)\\ &= \exp\left(\int_0^t [r_W(s) + \Psi(s)]ds\right).\end{aligned}$$

medRxiv preprint doi: https://doi.org/10.1101/2024.12.02.24318334; this version posted December 3, 2024. The copyright holder for this preprint (which was not certified by peer review) is the author/funder, who has granted medRxiv a license to display the preprint in perpetuity.
It is made available under a CC-BY 4.0 International license .

Supplementary Figures

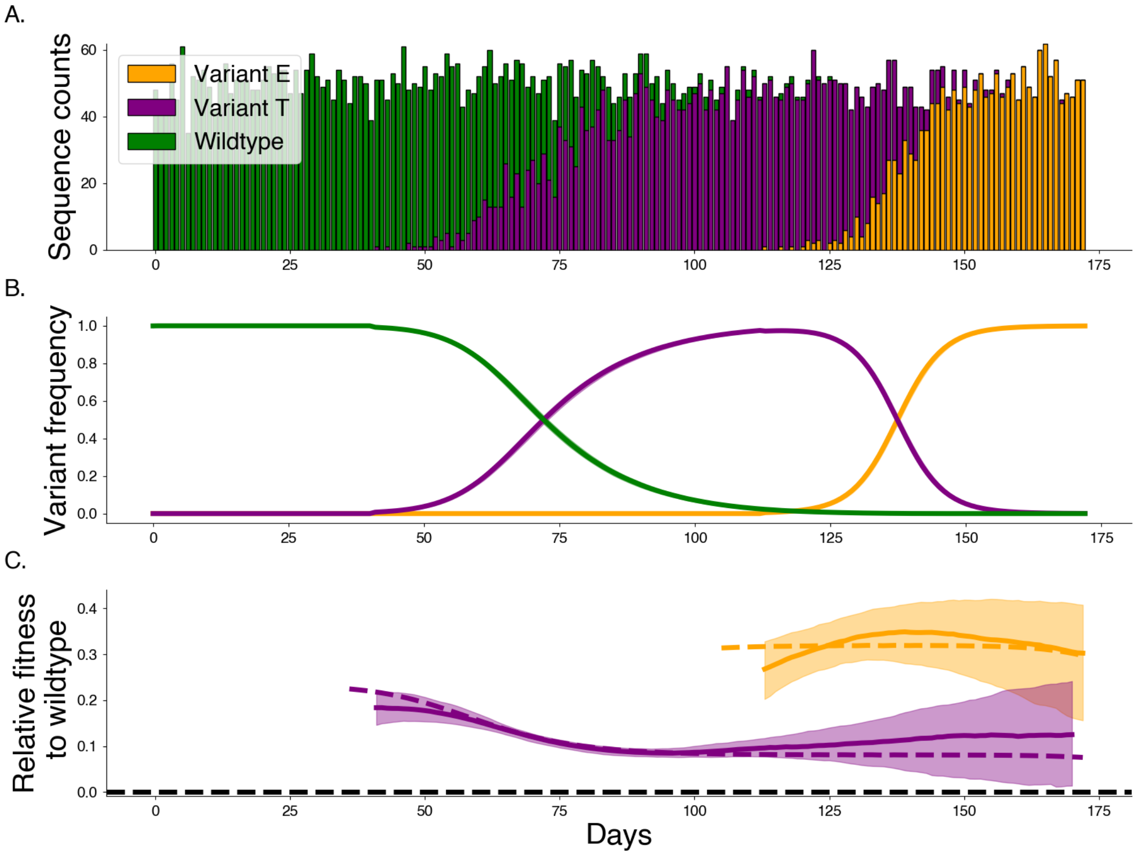

Fig. S1. Estimating relative fitness with Gaussian processes. Gaussian processes allow us a non-parametric estimate of the relative fitness for variants through time. This figure uses Gaussian processes to model the 3 variant example shown in Fig. 1. A. Synthetic sequence counts generated using a multinomial distribution with frequencies from Fig. 1C. B. Frequencies and posterior frequencies according to Gaussian process model. Intervals show the 80% credible interval. C. Relative fitnesses. Dashed line shows true relative fitnesses from underlying mechanistic model.

medRxiv preprint doi: https://doi.org/10.1101/2024.12.02.24318334; this version posted December 3, 2024. The copyright holder for this preprint (which was not certified by peer review) is the author/funder, who has granted medRxiv a license to display the preprint in perpetuity.
It is made available under a CC-BY 4.0 International license .

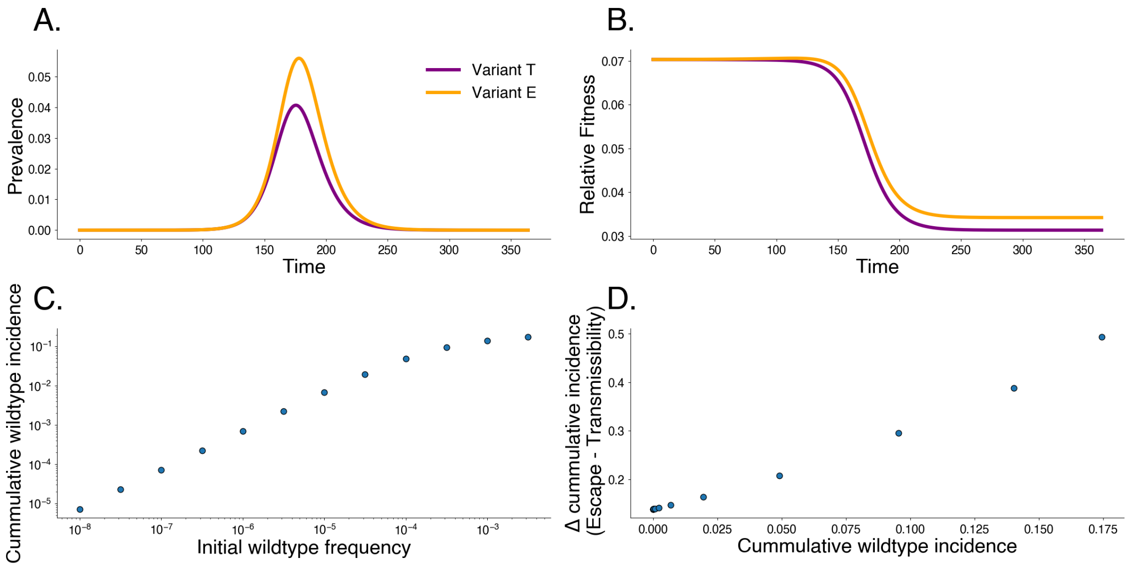

Fig. S2. Differences in fitness mechanisms impact frequency and prevalence in the short-term. Comparing simulations from two independent two-variant systems with either an escape variant E (orange) or a transmissibility variant T (purple). We fix the initial relative fitness for the two variants using Equation 2 and simulate dynamics for 365 days. (A) The prevalence for the variants. (B) The relative fitness from the variants. (C) The cumulative wildtype incidence as a function of the initial wildtype frequency. (D) The difference between the cumulative incidence between the escape variant and the transmissibility variant as a function of wildtype incidence.

medRxiv preprint doi: https://doi.org/10.1101/2024.12.02.24318334; this version posted December 3, 2024. The copyright holder for this preprint (which was not certified by peer review) is the author/funder, who has granted medRxiv a license to display the preprint in perpetuity.
It is made available under a CC-BY 4.0 International license .

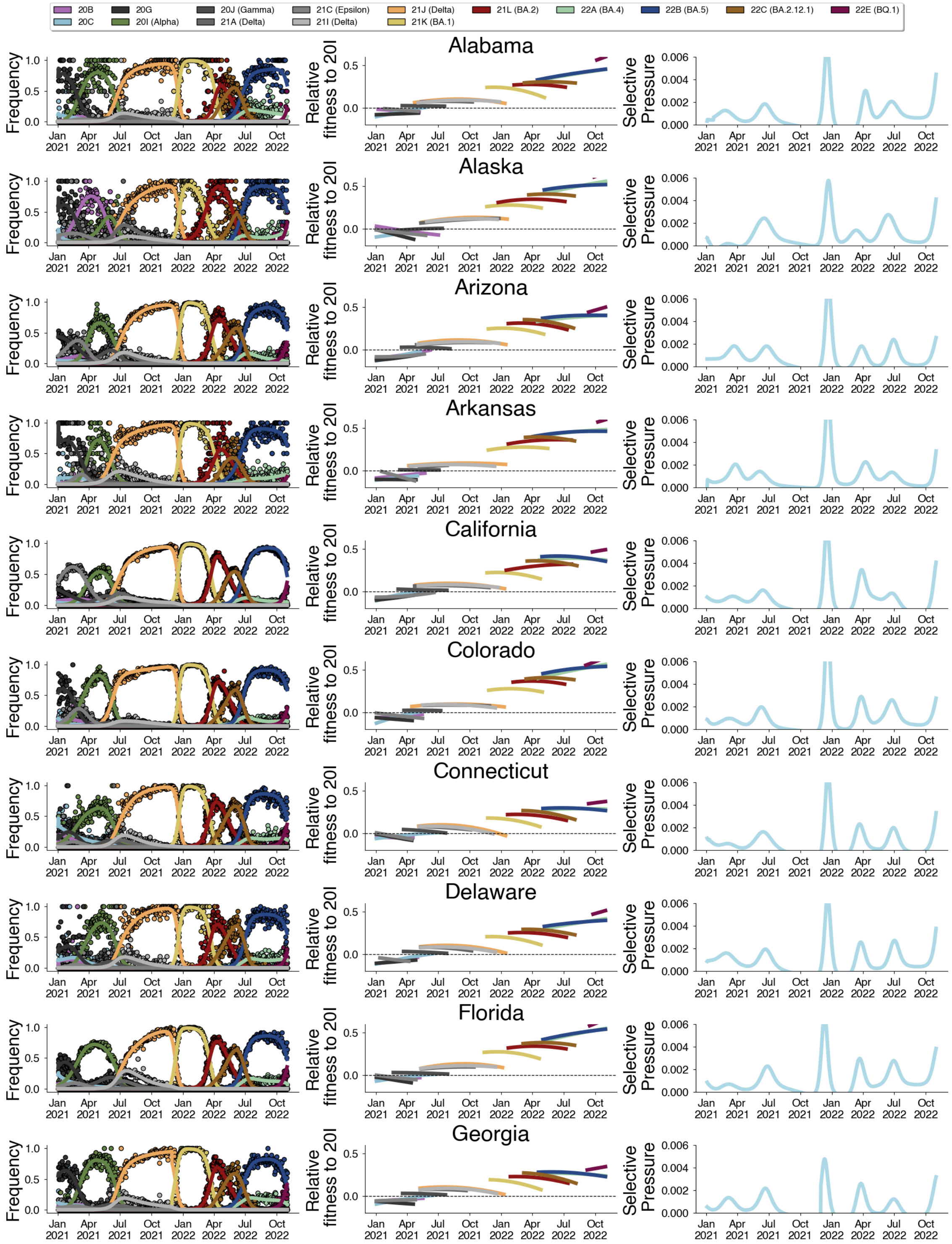

Fig. S3. Estimated variant frequencies, relative fitnesses, and selective pressure. Alabama through Georgia.

medRxiv preprint doi: https://doi.org/10.1101/2024.12.02.24318334; this version posted December 3, 2024. The copyright holder for this preprint (which was not certified by peer review) is the author/funder, who has granted medRxiv a license to display the preprint in perpetuity.
It is made available under a CC-BY 4.0 International license .

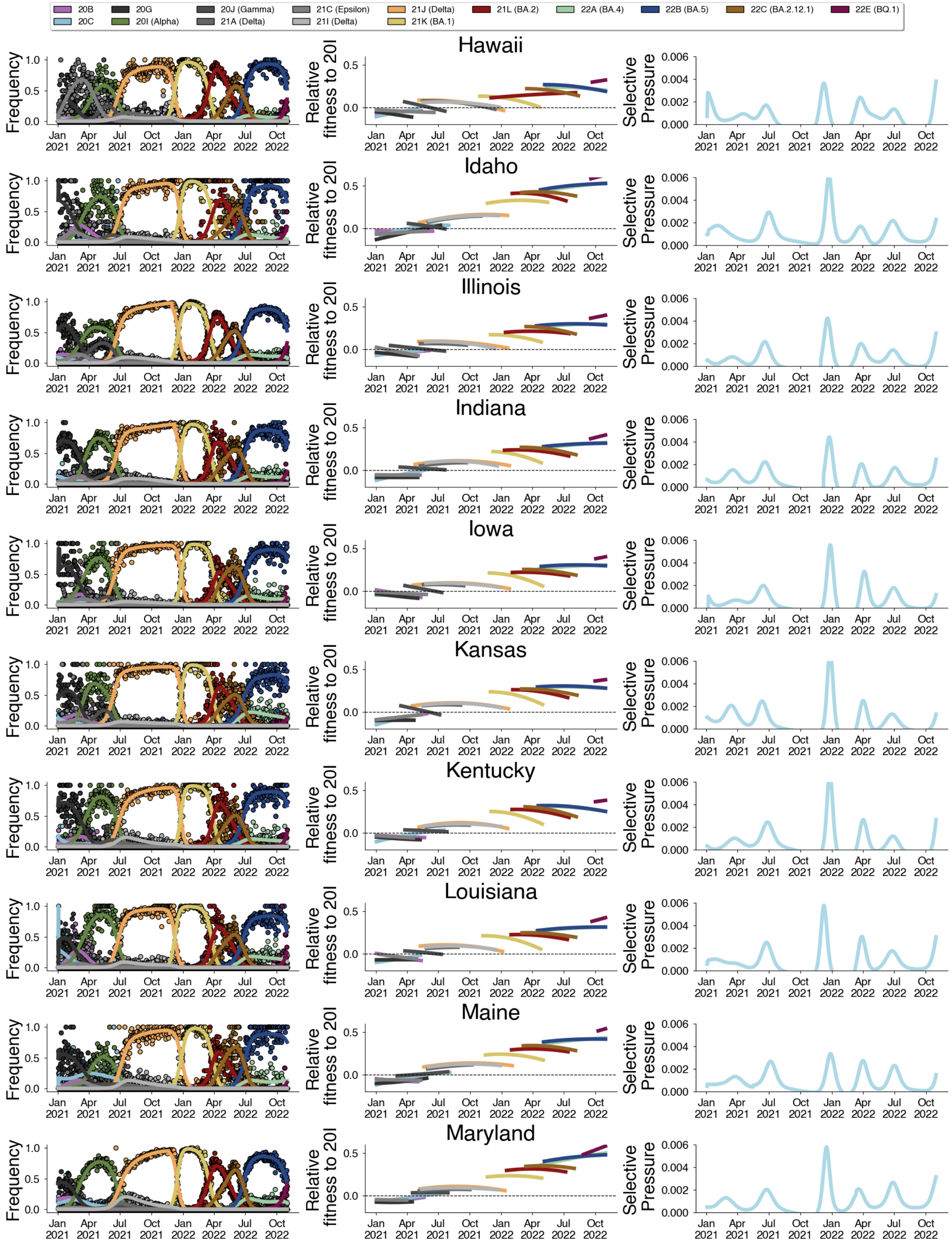

Fig. S4. Estimated variant frequencies, relative fitnesses, and selective pressure. Hawaii to Maryland.

medRxiv preprint doi: https://doi.org/10.1101/2024.12.02.24318334; this version posted December 3, 2024. The copyright holder for this preprint (which was not certified by peer review) is the author/funder, who has granted medRxiv a license to display the preprint in perpetuity.
It is made available under a CC-BY 4.0 International license .

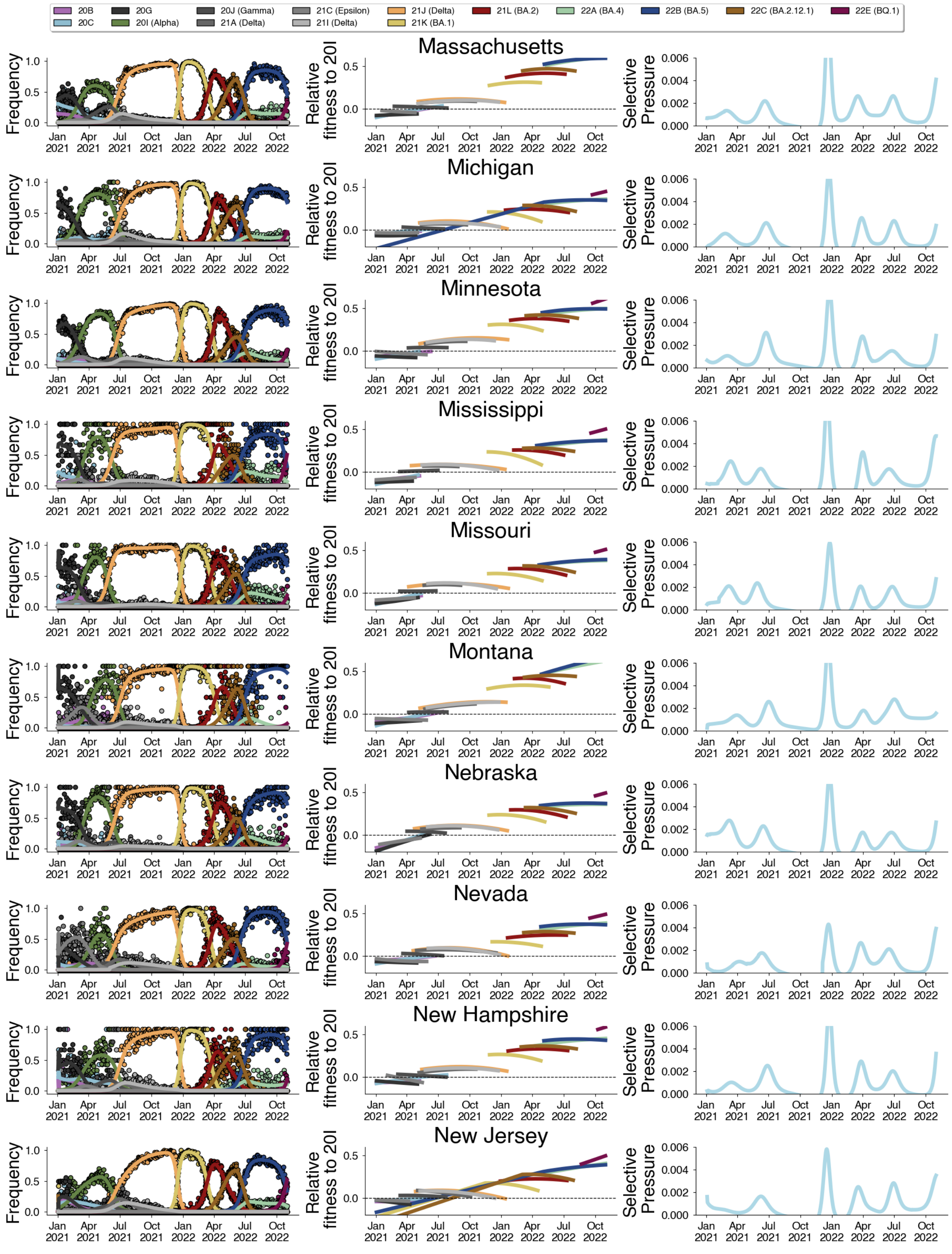

Fig. S5. Estimated variant frequencies, relative fitnesses, and selective pressure. **Massachusetts to New Jersey.**

medRxiv preprint doi: https://doi.org/10.1101/2024.12.02.24318334; this version posted December 3, 2024. The copyright holder for this preprint (which was not certified by peer review) is the author/funder, who has granted medRxiv a license to display the preprint in perpetuity.
It is made available under a CC-BY 4.0 International license .

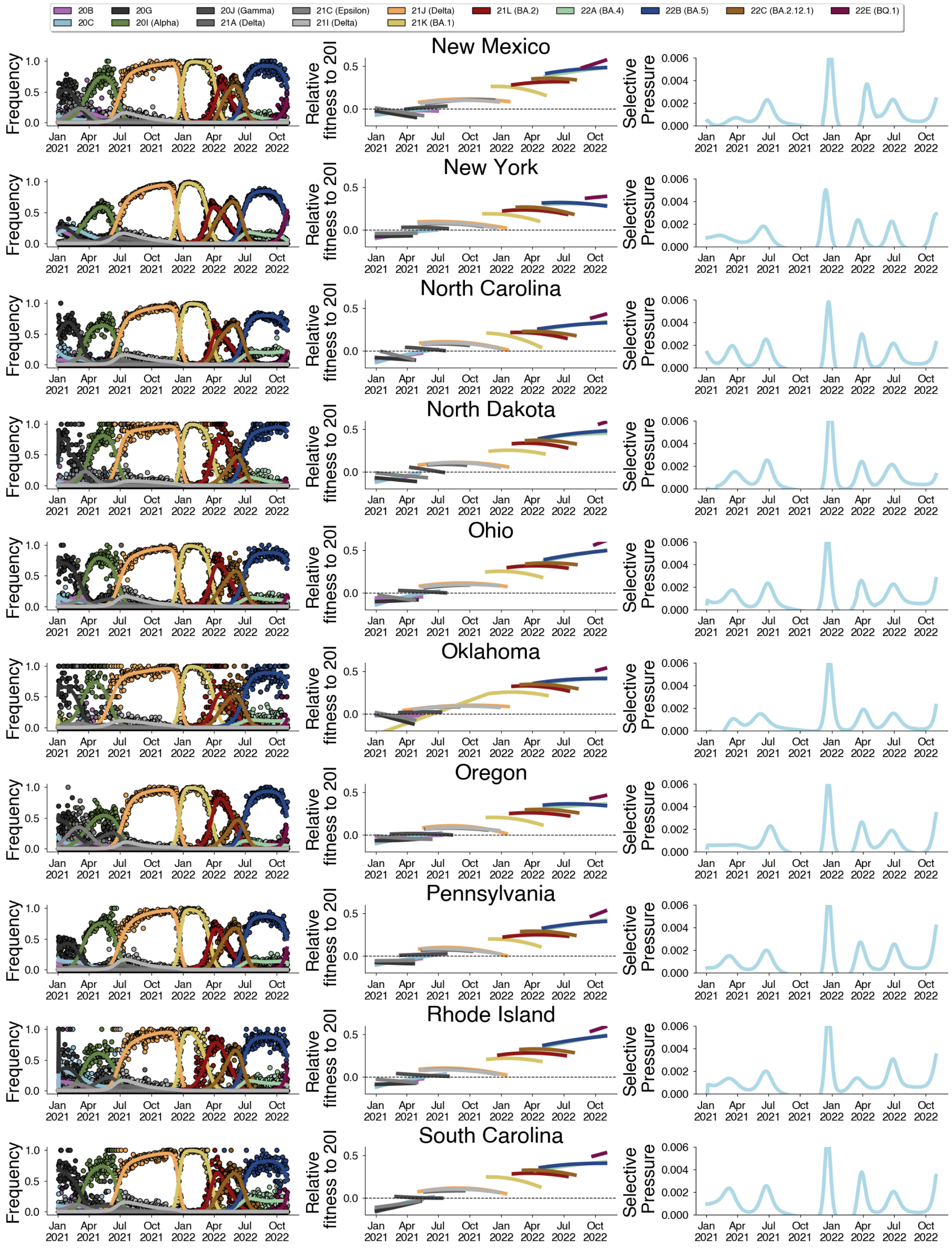

Fig. S6. Estimated variant frequencies, relative fitnesses, and selective pressure. New Mexico to South Carolina.

medRxiv preprint doi: https://doi.org/10.1101/2024.12.02.24318334; this version posted December 3, 2024. The copyright holder for this preprint (which was not certified by peer review) is the author/funder, who has granted medRxiv a license to display the preprint in perpetuity.
It is made available under a CC-BY 4.0 International license .

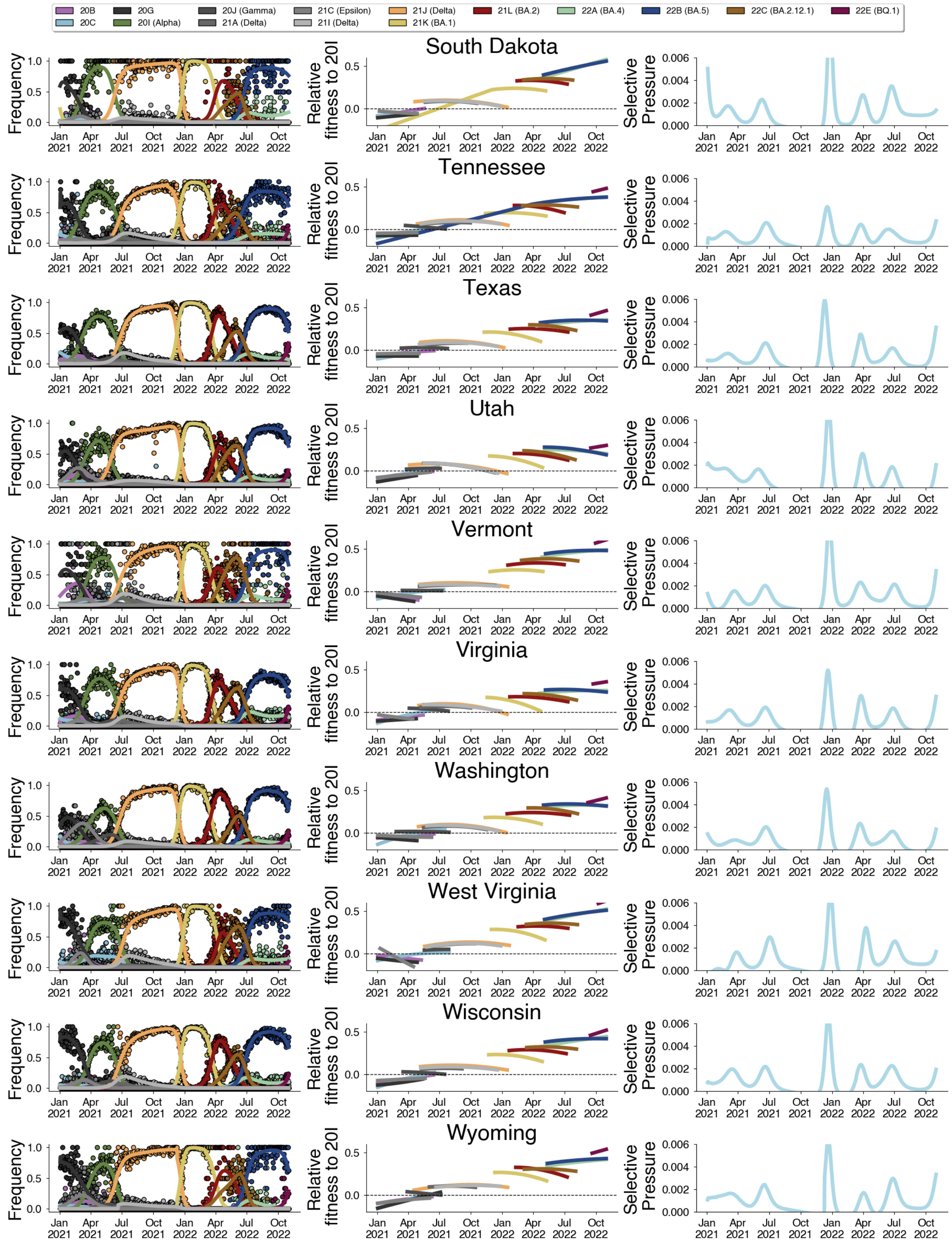

Fig. S7. Estimated variant frequencies, relative fitnesses, and selective pressure. South Dakota to Wyoming.

medRxiv preprint doi: https://doi.org/10.1101/2024.12.02.24318334; this version posted December 3, 2024. The copyright holder for this preprint (which was not certified by peer review) is the author/funder, who has granted medRxiv a license to display the preprint in perpetuity.
It is made available under a CC-BY 4.0 International license .

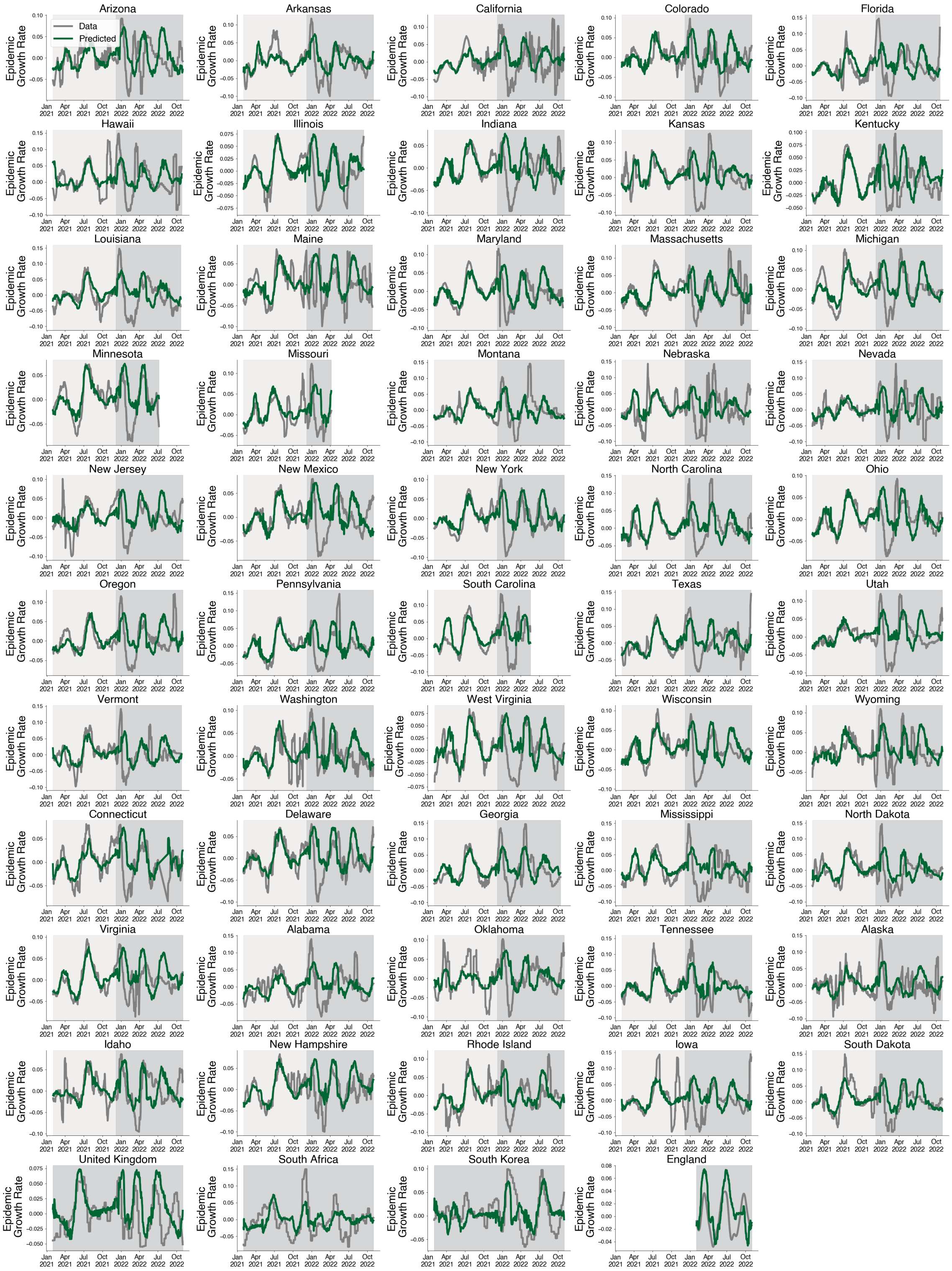

Fig. S8. Predictions for empirical growth rate using selective pressure for all locations.

medRxiv preprint doi: https://doi.org/10.1101/2024.12.02.24318334; this version posted December 3, 2024. The copyright holder for this preprint
(which was not certified by peer review) is the author/funder, who has granted medRxiv a license to display the preprint in perpetuity.
It is made available under a CC-BY 4.0 International license .

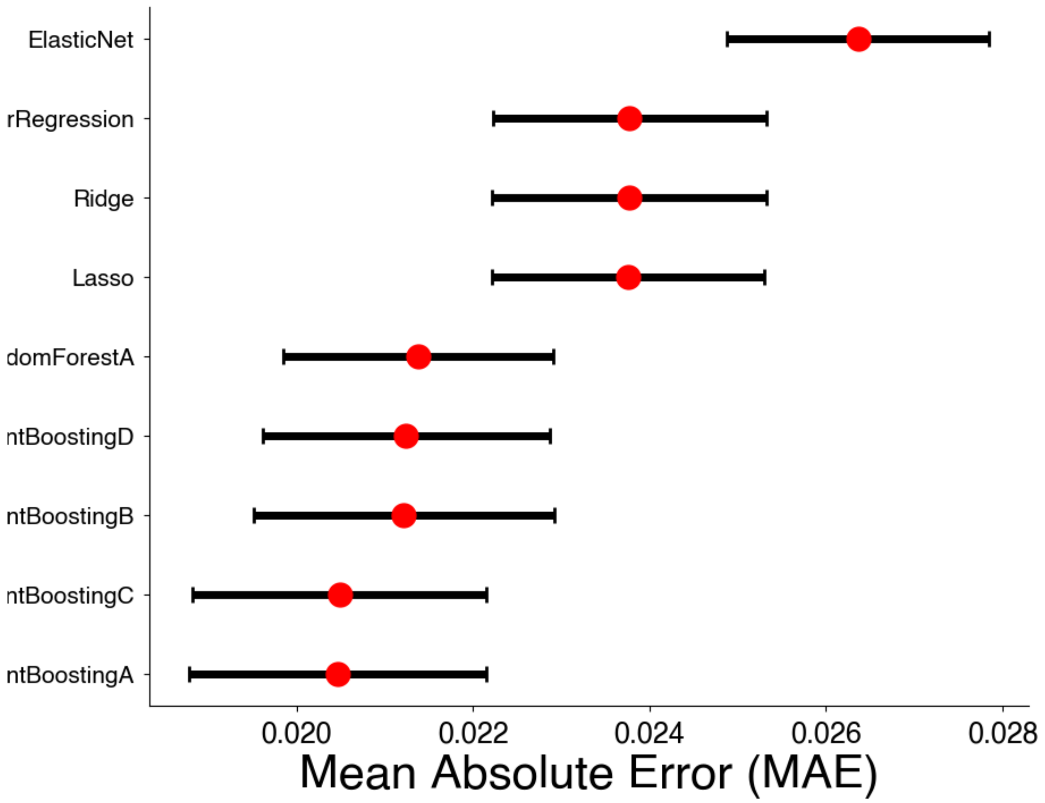

Fig. S9. Cross-validation error by model We compare the errors between models fit on 10 time series cross-validation splits.

medRxiv preprint doi: https://doi.org/10.1101/2024.12.02.24318334; this version posted December 3, 2024. The copyright holder for this preprint (which was not certified by peer review) is the author/funder, who has granted medRxiv a license to display the preprint in perpetuity.
It is made available under a CC-BY 4.0 International license .

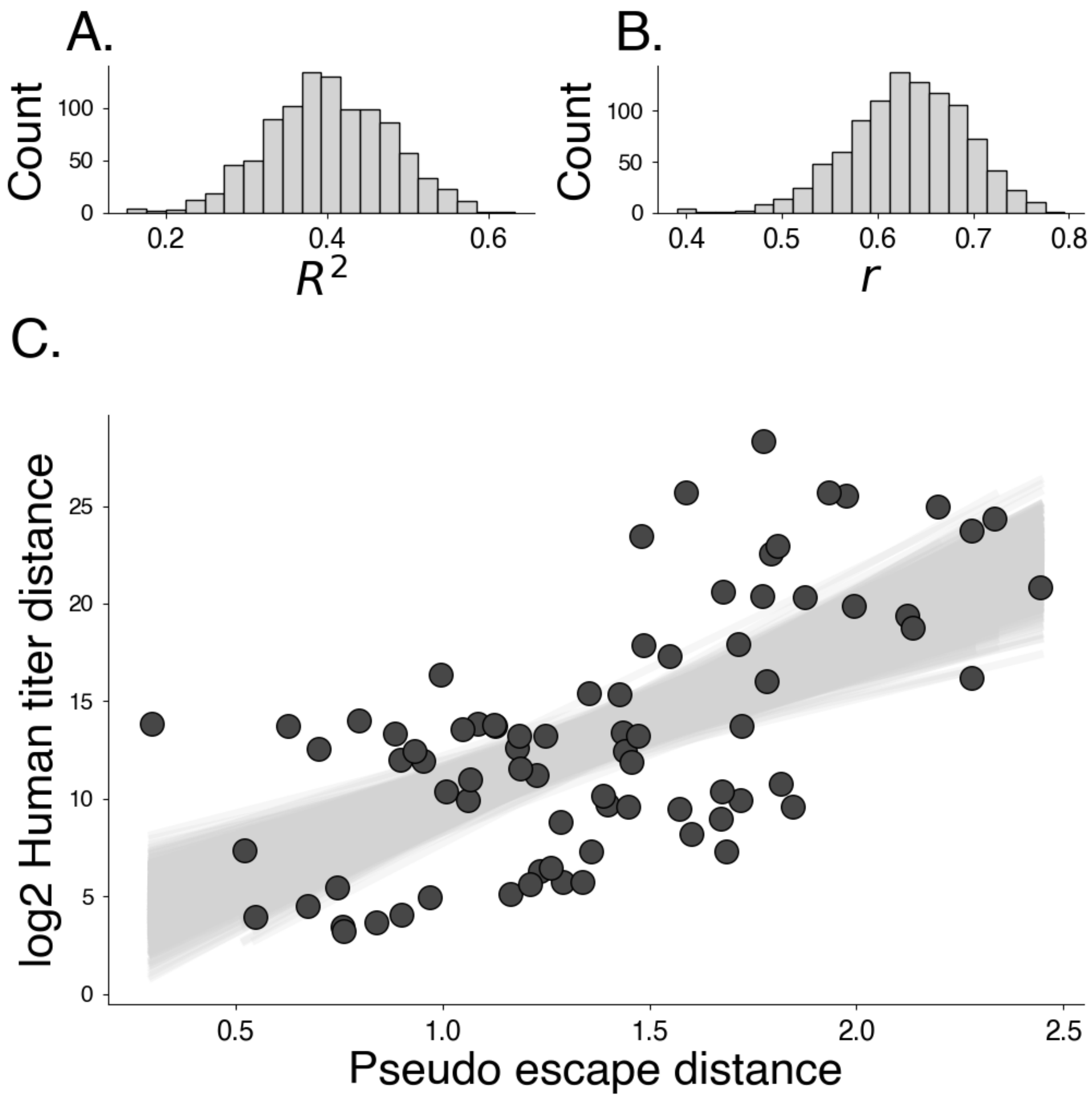

Fig. S10. Bootstrapping pseudo-immune distance and human titer distance analysis ($N_{\mathbf{replicate}} = 1,000$).

medRxiv preprint doi: https://doi.org/10.1101/2024.12.02.24318334; this version posted December 3, 2024. The copyright holder for this preprint (which was not certified by peer review) is the author/funder, who has granted medRxiv a license to display the preprint in perpetuity.
It is made available under a CC-BY 4.0 International license .

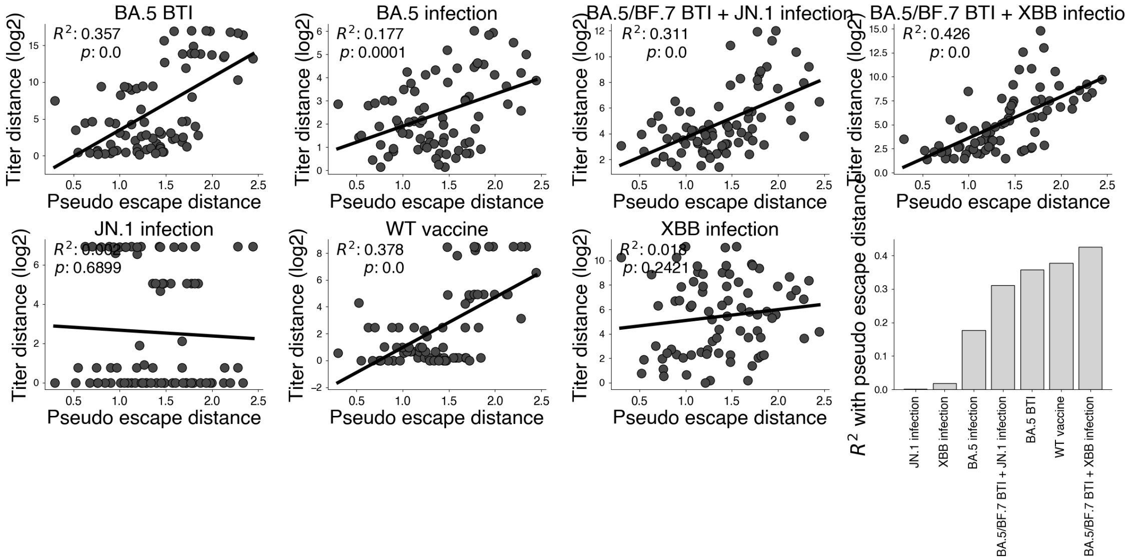

Fig. S11. Comparing pseudo-escape distance and titer distance between exposure groups.

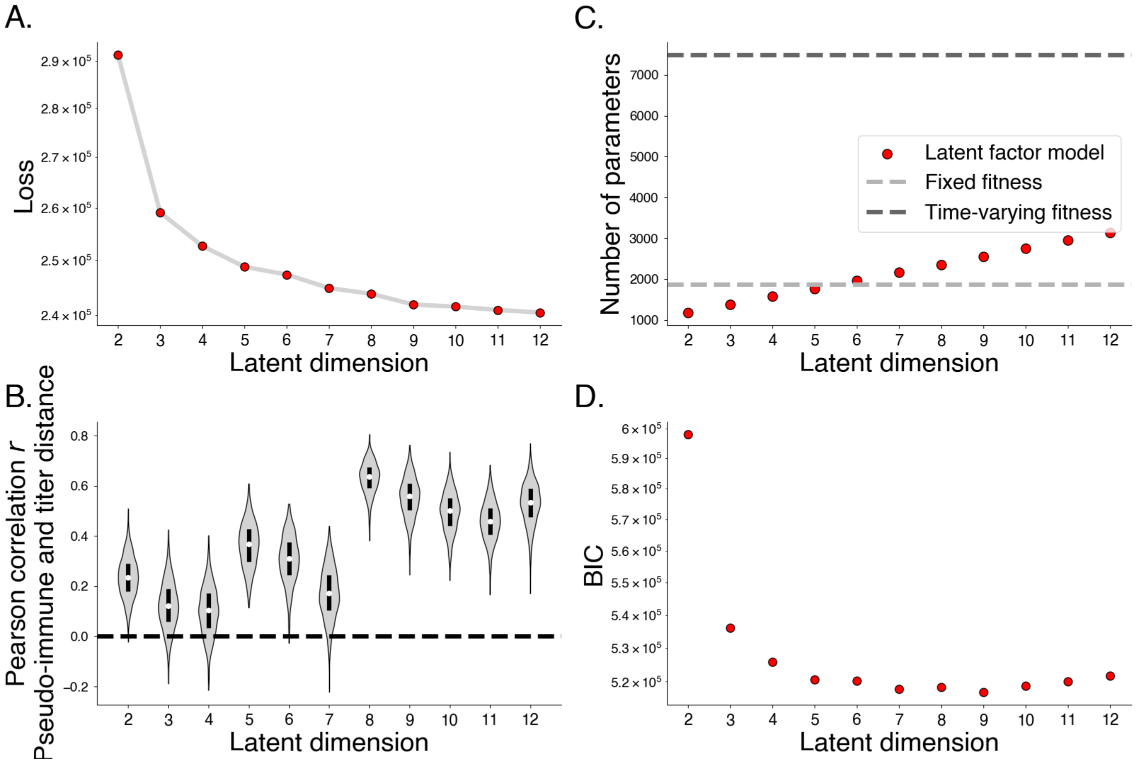

Fig. S12. Comparing latent factor model by number of latent immune dimensions. A. Maximum a posteriori loss by number of latent immune dimensions. B. Spearman correlation between pseudo-immune and titer distance by number of latent immune dimensions. C. Number of parameters by number of latent immune dimensions. D. Bayesian Information Criterion (BIC) by number of latent immune dimensions.

medRxiv preprint doi: https://doi.org/10.1101/2024.12.02.24318334; this version posted December 3, 2024. The copyright holder for this preprint (which was not certified by peer review) is the author/funder, who has granted medRxiv a license to display the preprint in perpetuity.
It is made available under a CC-BY 4.0 International license .

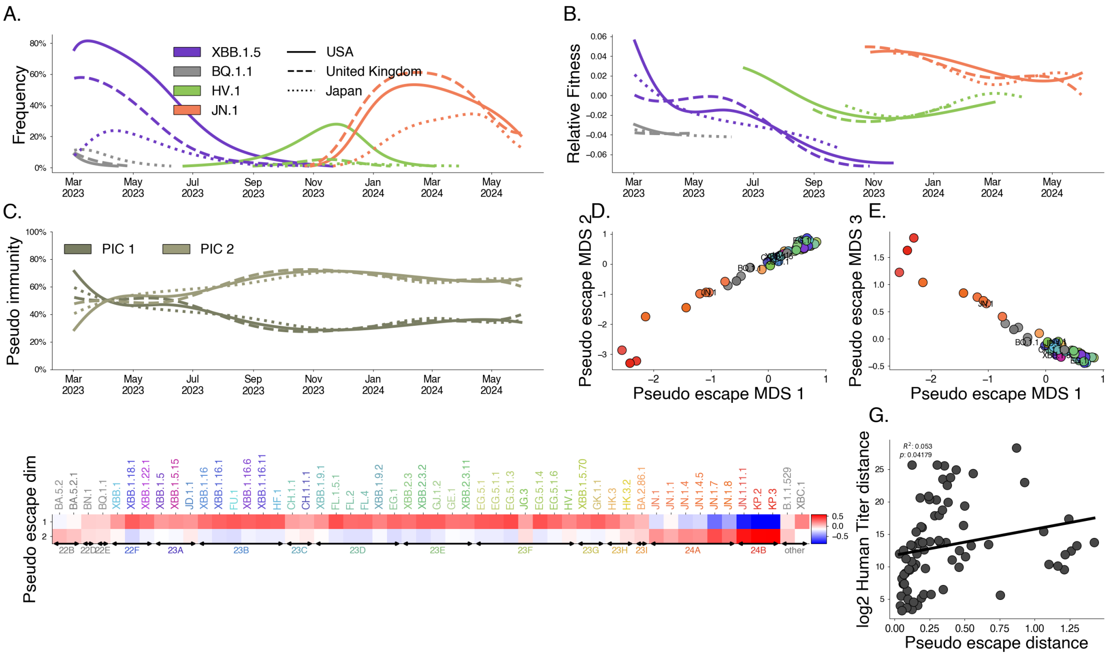

Fig. S13. Latent factor model with $D = 2$ pseudo immune dimensions.

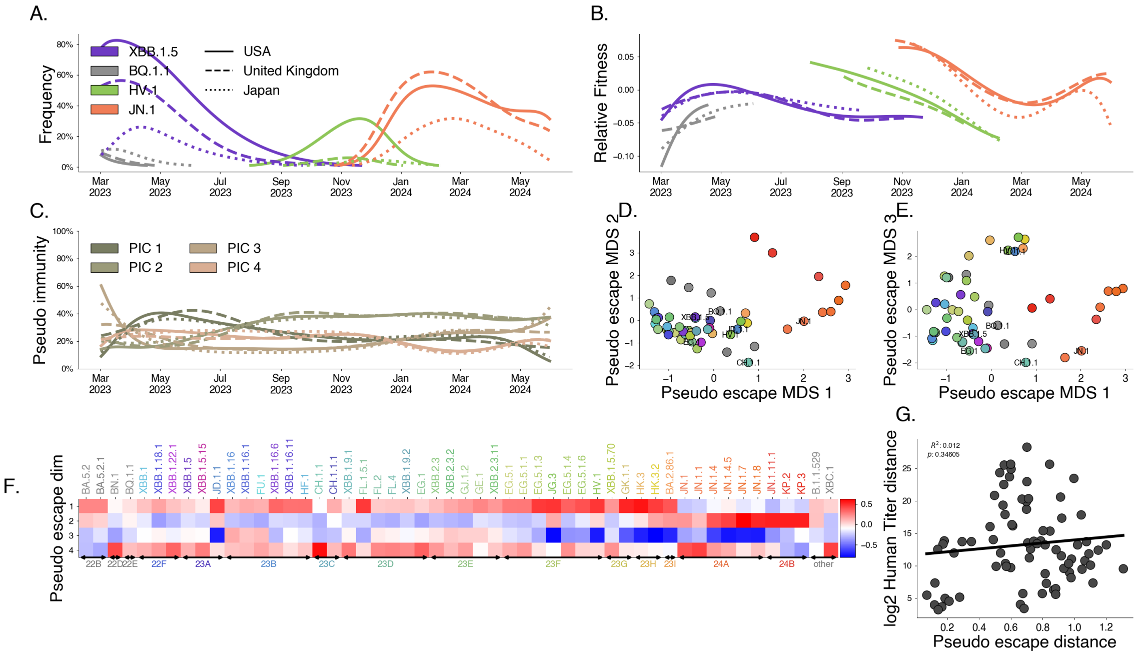

Fig. S14. Latent factor model with $D = 4$ pseudo immune dimensions.

medRxiv preprint doi: https://doi.org/10.1101/2024.12.02.24318334; this version posted December 3, 2024. The copyright holder for this preprint
(which was not certified by peer review) is the author/funder, who has granted medRxiv a license to display the preprint in perpetuity.
It is made available under a CC-BY 4.0 International license .

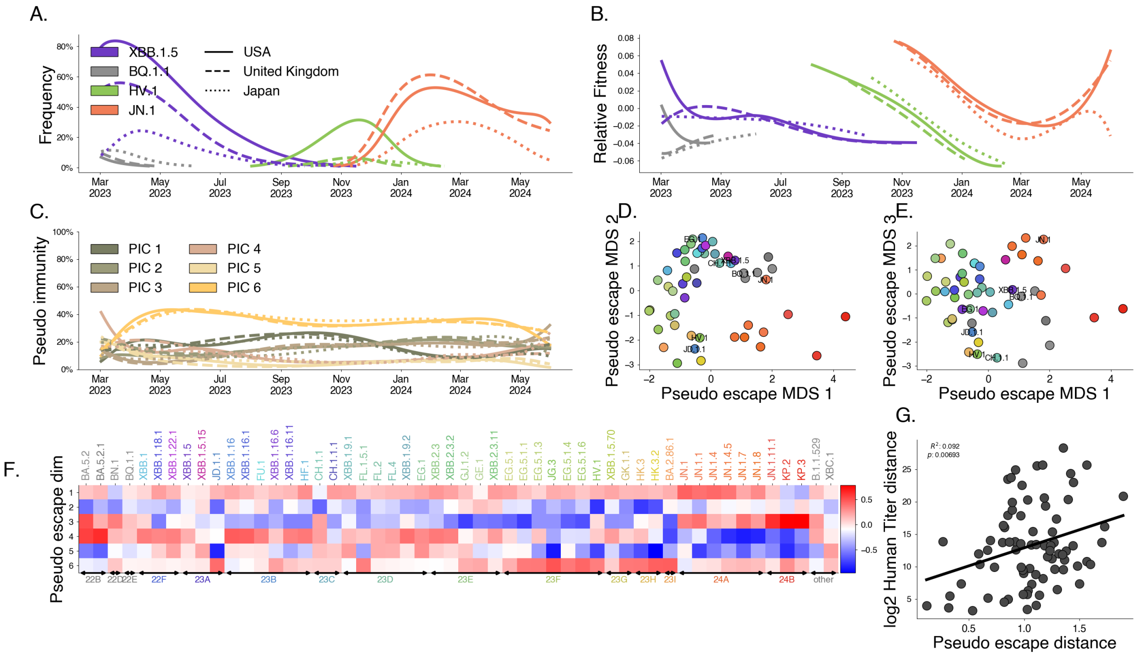

Fig. S15. Latent factor model with $D = 6$ pseudo immune dimensions.

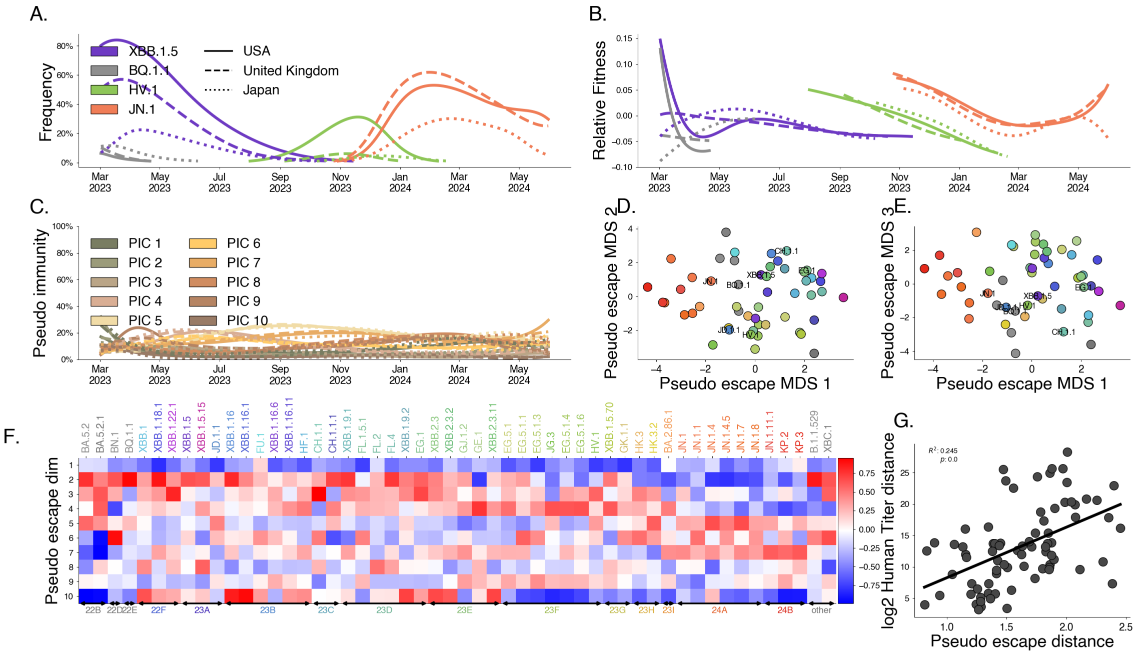

Fig. S16. Latent factor model with $D = 10$ pseudo immune dimensions.
